# Nano-orchestrated magnetotactic-like navigation for electromagnetic theranostics and immune enhancement *via* photoautotrophic oxygenation, mild hyperthermia, and ferroptosis

**DOI:** 10.1186/s12951-025-03488-7

**Published:** 2025-06-13

**Authors:** Chi-Hung Hsiao, Yung-Wei Lin, Chia-Hung Liu, Yan-Ting Chen, Hieu Trung Nguyen, Andrew E.-Y. Chuang

**Affiliations:** 1 Graduate Institute of Biomedical Materials and Tissue Engineering, College of Biomedical Engineering, Taipei Medical University, New Taipei City, 235603 Taiwan; 2 Department of Urology, Wan Fang Hospital, Taipei Medical University, 111 Hsing Long Road, Section 3, Taipei, 11696 Taiwan; 3 TMU Research Center of Urology and Kidney, Taipei Medical University, Taipei, 11031 Taiwan; 4 Department of Urology, School of Medicine, College of Medicine, Taipei Medical University, Taipei, 11031 Taiwan; 5 Department of Urology, Shuang Ho Hospital, Taipei Medical University, New Taipei City, 23561 Taiwan; 6 Department of Orthopedics and Trauma, Faculty of Medicine, University of Medicine and Pharmacy at Ho Chi Minh City, Ho Chi Minh City, 700000 Viet Nam; 7 International Ph.D. Program in Biomedical Engineering, College of Biomedical Engineering, Taipei Medical University, New Taipei City, 235603 Taiwan; 8 Cell Physiology and Molecular Image Research Center, Taipei Medical University-Wan Fang Hospital, Taipei, 11696 Taiwan; 9 Precision Medicine and Translational Cancer Research Center, Taipei Medical University Hospital, Taipei, 11031 Taiwan

**Keywords:** Magnetotactic-like system, Electromagnetic theranostic, Immune augmentation, Photoautotrophic oxygenation

## Abstract

Overcoming the hypoxic tumor microenvironment (TME) and immune suppression remains a significant challenge in solid bladder tumor therapies. This study introduces a translational system of nano-orchestrated magnetotactic-like system, integrating photosynthetic oxygenation, remote hyperthermia, and ferroptosis to achieve comprehensive tumor eradication and immune activation. The developed system, composed of electromagnetic-responsive iron oxide nanoparticles (IO NPs) encapsulated within a glycol chitosan (GCS) matrix and coated onto *Chlorella* (CHL; CHL-GCS-IO NPs), exhibited versatility for precise magnetic targeting, photothermal-hypertehrmia and photosynthesis-driven oxygen generation under light irradiation. The CHL enhanced oxygen production by continuously alleviating hypoxia, boosting both electromagnetic therapeutic efficacies and ferroptosis-induced tumor cell death. Moreover, the multimodal CHL-GCS-IO NPs reprogrammed the TME, facilitating immune activation by promoting macrophage polarization towards the proinflammatory M1 phenotype, engaging cytotoxic T cells and natural killer cells, programmed death ligand 1 (PD-L1) downregulation, and driving dendritic cell reprogramming towards improved antigen presentation. In vivo, this approachsuggested significant tumor growth inhibition and prevented recurrence in bladder cancer models, highlighting its potential for robust and durable anticancer immunity. This magnetotactic-like CHL platform presents a highly promising theranostic strategy, merging multimodal therapies with immune modulation to tackle both direct and systemic challenges of solid bladder tumors.

**Graphical abstract:**

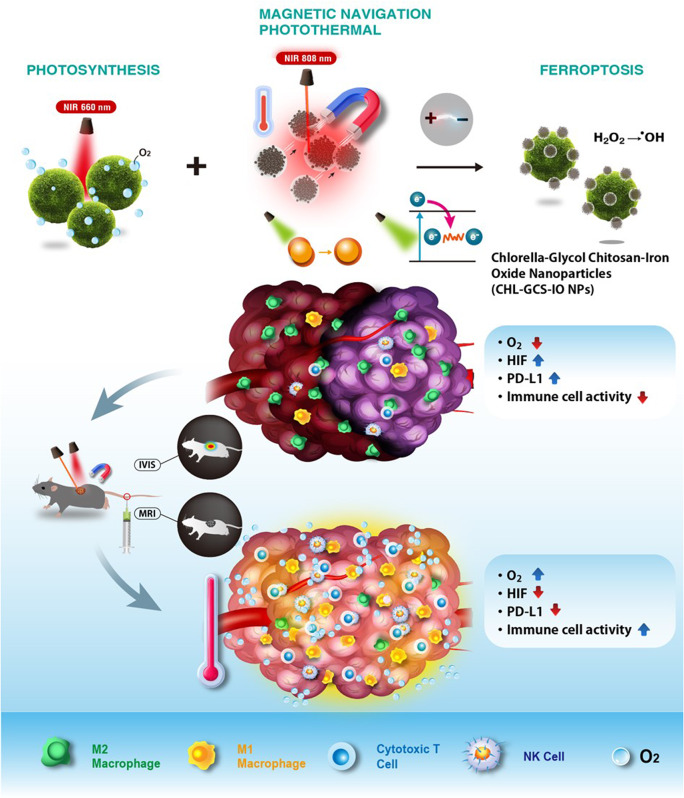

**Supplementary Information:**

The online version contains supplementary material available at 10.1186/s12951-025-03488-7.

## Introduction

The solid tumor microenvironment (TME) is highly complex, largely due to the rapid proliferation of tumor cells, abnormal growth of the tumor vasculature, and altered energy metabolism [1]. These characteristics significantly limit the effectiveness of cancer treatments [2–5]. While numerous drugs and therapeutic strategies have emerged in recent decades, chemotherapy remains a primary and widely used treatment for many malignant tumors [6–9]. However, current chemotherapy protocols often fail to deliver optimal outcomes. The primary cause of treatment failure in over 90% of cancer patients is drug resistance, which is driven by the unique characteristics of the TME [10–14]. To counteract this resistance, higher drug dosages are frequently administered, leading to severe adverse effects toward healthy tissues. Thus, there is a critical need for alternative strategies that disrupt the TME, reverse drug resistance, and promote greater drug accumulation within tumor cells. Such as approach would not only improve the efficacy of chemotherapy but also reduce its side effects.

A common characteristic of most solid tumors is the presence of hypoxia within the TME [15], which significantly contributes to resistance against anticancer therapies [19–26]. Hypoxia induces upregulation of hypoxia-inducible factor (HIF)-1α, promoting angiogenesis and the polarization of M2 macrophages [27], both of which play key roles in driving chemotherapy resistance. Moreover, recent research demonstrated that hypoxia also reduces tumor cell sensitivity to chemotherapeutic agents. As a result, overcoming hypoxia-induced resistance represents a critical target for improving cancer treatment outcomes [28].

The hypoxic TME also plays a crucial role in promoting the differentiation of immune-suppressive stromal cells and increasing the production of immunosuppressive molecules, such as checkpoint inhibitors, while also altering the tumor’s metabolic landscape [29]. These factors contribute to tumor immune tolerance, which is a major challenge limiting the effectiveness of clinical immunotherapies for treating solid tumors [30]. Additionally, hypoxia in the tumor environment reduces the generation of reactive oxygen species (ROS), thereby diminishing the efficacy of clinical oncological therapies [28].

To address tumor hypoxia, various strategies are being developed, including the use of hemoglobin or perfluorocarbons for direct oxygen delivery, and catalyzing the conversion of H_2_O_2_ into O_2_ using biomedical materials such as MnO_2_, CaO_2_, platinum (Pt), and catalase, which transiently raise oxygen levels within tumors. While these methods temporarily increase oxygen levels and improve the antitumor effects of dynamic therapies, their limited dosage and short therapeutic windows might not sufficiently counteract the hypoxic environment or fully reverse the associated immunosuppression [33].

Developing a method to create a sustained, biocompatible, and efficient oxygen-generating system for tumor therapy is of significant interest. Microalgae offer several advantages for cancer treatment, such as ease of cultivation, low cost, and intrinsic tumor-targeting properties [34, 35]. Numerous studies have explored the tumor-targeting capabilities of microorganism for precise tumor suppression, including the targeted delivery of drugs or functional nanoparticles (NPs). Photosynthetic microalgae, with their photoautotrophic abilities, utilize chlorophyll molecules within their thylakoid membranes to drive oxygen-producing photosynthesis. The ability to control their own growth and photosynthetic activity using light makes them particularly attractive for therapeutic applications [37]. *Chlorella* (CHL), a type of natural photosynthetic microalgae, played a key role in oxygen production during the evolution of life on Earth. Its oxygen-evolving properties have been explored for treating cardiovascular diseases, although its application in biological bladder tumor oxygenation for cancer therapy remains underexplored. When combined with phototherapy, ferroptosis can enhance anti-tumor immunity by promoting the release of tumor-associated antigens, triggering immunogenic cell death [38]. Traditional cytokine therapies, while effective, often lead to systemic activation of non-specific T cells, resulting in potential toxicity. A targeted approach that integrates localized cytokine signaling with tumor antigen presentation could improve safety and efficacy, leading to stronger and more specific immune responses against cancer [39].

Given their potential to alleviate tumor hypoxia and superiority to photodynamic therapy (PDT) [40], photosynthetic microalgae like CHL represent a promising approach for tumor treatment. Over years of research, various types of microalgal cells have been developed as delivery vehicles for chemotherapeutic drugs, plasmids, proteins, and other nanomedicines in cancer therapy [37]. These composite nanosystems enable microalgal cells to perform multiple therapeutic functions, allowing for a comprehensive, multimodal approach to treatment, including Fenton noncatalytic therapy, photothermal ablation, and biological therapy. CHL holds the potential to reduce tumor immunosuppression by continuously regulating the hypoxic TME. Through light-driven photosynthesis, this approach—known as photosynthesis therapy (PST)—can enhance the effectiveness of dynamic cancer therapies. However, as an exogenous microorganism, CHL could elicit an immune response following an intravenous (*i.v.*) injection, leading to its clearance before it can accumulate in tumor tissues. A key challenge for the in vivo application of CHL is to enhance its theranostic functionality, retention, and targeted delivery to tumors.

Minimally invasive surgery and targeted therapies aided by the concept of microrobots are innovative approaches that minimize the level of intervention, focus drug delivery to specific locations, and reduce the risk of adverse side effects. These untethered microrobots have the ability to navigate into small, hard-to-reach areas in a noninvasive manner, offering clinicians new tools to address diseases requiring access to difficult regions of the body [41]. For controlled in vivo use, microrobots must overcome several challenges: they must be able to swim under external stimuli such as magnetic fields, chemical gradients, ultrasound, magnetoacoustic signals, electric fields, or light; they must achieve high contrast during medical imaging; they must successfully transport therapeutic cargo to target sites; and they must be biocompatible. The biohybrid approach leverages the motility of natural cells like sperm, algae, or bacteria, as well as the contractile force of muscle cells, to generate movement. Similarly, biohybrid microrobots, powered by flagella, exhibit behaviors such as pH-taxis, aerotaxis, and chemotaxis [42].

**Fig 1:**
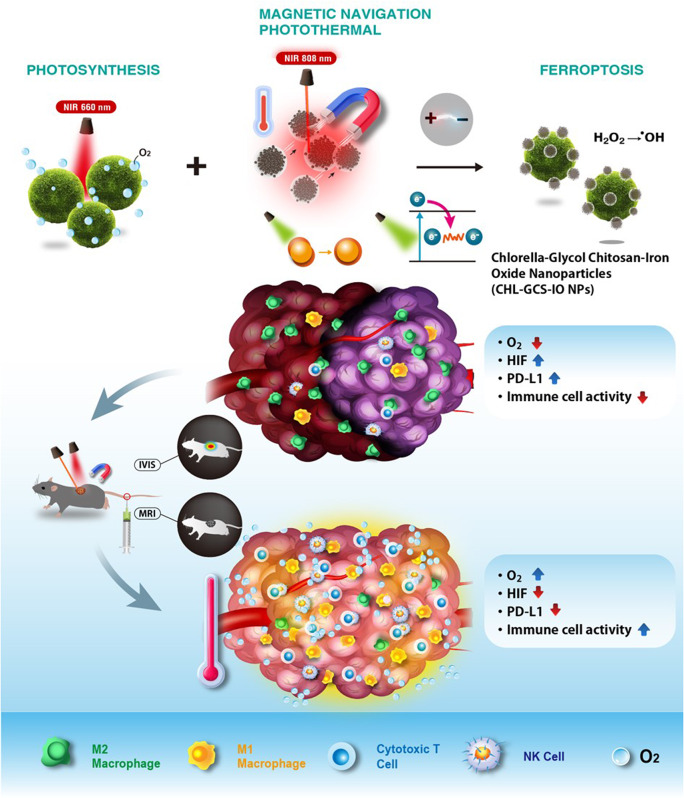
Schematic illustration of the nano-orchestrated magnetotactic-like system (*Chlorella* (CHL)-glycol chitosan (GCS)-iron oxide (IO) nanoparticles (NPs)) for multimodal cancer therapy. The system integrates photosynthetic oxygenation, photothermal therapy (PTT), and ferroptosis to modulate the tumor microenvironment (TME), alleviate hypoxia, and activate immune responses. The magnetotactic-likesystem were designed to be guided by an external magnetic field, enabling targeted delivery to bladder tumors while enhancing propulsion and tumor accumulation. Once within the TME, the CHL-GCS-IO NPs created sustained oxygen production, reversing hypoxia and boosting immune activation by engaging T cells, macrophages, natural killer (NK) cells, and dendritic cells (DCs). This comprehensive therapeutic approach aimed to induce cancer cell death via ferroptosis and photothermal effects, inhibit tumor recurrence, and establish antitumor immune memory, potentially preventing future bladder cancer relapse

Herein, we developed a drug-free magnetotactic-like CHL system by incorporating electromagnetic-responsive iron oxide (IO) into glycol chitosan (GCS) NPs coated with CHL (CHL-GCS-IO NPs) to enhance the theranostic efficacy, promote efficient tumor accumulation, and reduce clearance, as depicted in Fig. 1. We hypothesize that the nano-orchestrated magnetotactic-like system (CHL-GCS-IO NPs) can overcome the limitations of conventional bladder cancer therapies by integrating precise magnetic targeting, photosynthetic oxygenation, electromagnetic hyperthermia, and ferroptosis-induced tumor cell death. We propose that sustained oxygenation from Chlorella (CHL) will alleviate hypoxia, enhancing reactive oxygen species (ROS) production and ferroptosis efficacy. This, in turn, will modulate the tumor microenvironment (TME), promoting macrophage polarization toward the M1 phenotype, PD-L1 downregulation, and dendritic cell activation, leading to robust anticancer immunity. Additionally, the incorporation of glycol chitosan (GCS) ensures biocompatibility and controlled ROS generation, while magnetic guidance enhances tumor targeting, minimizing systemic toxicity. By combining these mechanisms, we anticipate that this multimodal theranostic approach will not only inhibit tumor growth but also prevent recurrence through sustained immune activation.

This system was designed to facilitate the targeted delivery of CHL-GCS-IO NPs, enabling magnetic actuation while maintaining flexibility for propulsion within the bladder TME. Alleviating hypoxia and boosting immune activation were aimed at improving the efficacy of electromagnetic theranostics. Magnetic actuation drives CHL-GCS-IO NPs propulsion in vivo, while IO NPs enhance ferroptosis of bladder tumor cells. Furthermore, the sustained oxygen production from CHL-GCS-IO NPs is expected to continuously mitigate hypoxia-related features of the tumor and reverse the immunosuppressive microenvironment. The combined effects of electromagnetic therapy and ferroptosis are anticipated to induce cancer cell death and inhibit tumor recurrence. Additionally, this approach is projected to activate T cells, natural killer (NK) cells, and dendritic cells (DCs), engaging the programmed death ligand 1 (PD-L1) pathway, which could contribute to robust antitumor immune memory, providing resistance against future bladder cancer recurrence.

## Experimental section

### Materials

GCS (≥ 60% titration, crystalline), Dihydroethidium (DHE) Assay Kit, CCK-8, 4’,6-diamidino-2-phenylindole (DAPI), fluorescein isothiocyanate (FITC), and FeCl_3_∙6H_2_O were obtained from Sigma-Aldrich (St. Louis, MO, USA). Iron (II) chloride (FeCl_2_, anhydrous, 99.5%) was sourced from Alfa Aesar (Ward Hill, MA, USA). RAW264.7 cells were purchased from the American Type Culture Collection (ATCC, Manassas, VA, USA), and MB49 cells were acquired from Merck (Darmstadt, Germany). Antibodies targeting cluster of differentiation 86 (CD86, M1 macrophages), CD206 (M2 macrophages), CD8^+^ (cytotoxic T cells), HIF-1α, PD-L1, CD49b, CD11c, and TGF-β were supplied by Asia Bioscience (Taipei, Taiwan). Notably, all chemicals were used without further purification. Phosphate-buffered saline (PBS) solutions were prepared using 20 mM phosphate buffer and 0.1 M NaCl, adjusted to pH 7.4. All solutions were prepared with filtered and ultraviolet-treated ultrapure water with a resistivity of 18.2 MΩ∙cm. *Chlorella* (CHL) was obtained from Shopee (Taiwan). For the experimental procedures, light-emitting diode (LED) lights at wavelengths of 660 (10 min at 0.1 W/cm²) and 808 nm (5 min at 2.45 W/cm²) were used.

### Fabrication and analysis of GCS-IO NPs

GCS-IO NPs were prepared using a modified co-precipitation method. GCS was dissolved in ultrapure water to form a homogeneous solution. Separately, FeCl_2_ (244 mg) and FeCl_3_∙6H_2_O (525 mg) were dissolved in water under an inert atmosphere. The GCS solution (4 mg/ml) was then slowly added to the iron chloride mixture with continuous stirring. The solution was adjusted to pH 10 using NaOH to initiate the co-precipitation process, forming IO NPs within the GCS matrix. The resulting GCS-IO NPs were collected by centrifugation, washed with ultrapure water to remove impurities, and freeze-dried for further use.

The GCS-IO NPs were characterized using various analytical techniques. Dynamic light scattering (DLS) was employed to measure the particle size and zeta potential, confirming the nanoscale size and stability of the NPs in solution. Transmission electron microscopy (TEM) was used to observe the morphology and core-shell structure of the GCS-IO NPs. The presence of IO within the NPs was verified by x-ray diffraction (XRD), confirming the crystalline nature of the IO core. Fourier-transform infrared (FTIR) spectroscopy was used to analyze the chemical bonding and confirm the successful encapsulation of the IO within the GCS matrix.

Optimization of GCS-IO NPs involved a series of characterization techniques to evaluate their properties. The mean hydrodynamic diameter and zeta potential of the NPs were measured at 25 °C using dynamic light scattering (DLS) with a Malvern Zetasizer Nano-ZS90 (Malvern, Worcestershire, UK) to assess the particle size distribution and surface charge.

To examine the morphology and structural features of the NPs, transmission electron microscopy (TEM) was performed using a Hitachi HT-7700 (Tokyo, Japan). Fourier-transform infrared (FTIR) spectroscopy, conducted with a Nicolet™ iS™ 10 spectrometer (ThermoFisher Scientific, Waltham, MA, USA), was employed to analyze the chemical bonding of the GCS-IO NPs. Spectra were recorded at room temperature with a resolution of 4 cm^− 1^ over a range of 4000 to 500 cm^− 1^. In addition, an XRD analysis was performed using a Bruker D2 PHASER (Billerica, MA, USA) to assess the crystalline structure of the NPs. In addition to the aforementioned techniques, the magnetic properties of the GCS-IO NPs were assessed using a superconducting quantum interference device (SQUID) magnetometer. This analysis provided detailed information on the magnetic behavior of the NPs, such as their magnetization curves and magnetic susceptibility, allowing for precise measurement of their responsiveness to external magnetic fields. For the experiment, a linear coil with a diameter of ≈ 2.2 cm was used, along with a 50–60 Hz mains transformer serving as an AC electromagnet. The system was powered by a 110–240 V supply.

The photothermal properties of PBS, IO NPs, and GCS-IO NPs were evaluated by subjecting them to near-infrared (NIR) irradiation using an 808-nm laser with a power density of 2.45 W/cm² while in an aqueous dispersion. Temperature changes of the samples were recorded over a specified time period. The photothermal conversion efficiency was calculated as the ratio of the internal energy increase in the fluid to the total incident light radiation.

The formula used to determine the photothermal conversion efficiency was [heat capacity of the fluid (4.2 J/(g/°C)) × mass of fluid (g) × increase of average temperature (°C)] / [time of irradiation (s) × density of power of NIR (W/cm^2^) × irradiated zone (cm^2^)] × 100. This formula allowed quantification of the efficiency with which the NPs converted the absorbed NIR light into heat, a critical parameter for assessing their suitability for PTT applications.

### Fabrication and analysis of CHL-GCS-IO NPs

To load GCS-IO NPs onto CHL, a suspension of CHL (10^7^ cells/mL) was mixed with various concentrations of GCS-IO NPs (0, 5, 50, 125, 250, and 500 µg) and incubated at 25 °C for 30 min. The CHL-GCS-IO NPs were then separated from any unbound GCS-IO NPs by centrifugation and subsequently washed with PBS. Optical microscopy (Leica, Wetzlar, Germany) was employed to examine the morphology of the CHL-GCS-IO NPs, while fluorescence spectra were recorded with a fluorescence spectrometer (Spark^®^ multimode microplate reader, Tecan Group, Männedorf, Switzerland). Zeta potentials of both CHL and CHL-GCS-IO NPs were determined using DLS with a Malvern Zetasizer Nano-ZS90.

In this study, we investigated the biodistribution of GCS-IO NPs both in vitro and in vivo, as well as potential reproductive biological mechanisms. For bioimaging purposes, FITC was conjugated to the NPs through amide bonds, enabling tracking in both in vitro and in vivo experiments. Fluorescence microscopy was used to detect the biodistribution of FITC-GCS-IO NPs (green fluorescence) and the natural chlorophyll of CHL (red fluorescence) in CHL and CHL-FITC-GCS-IO NPs samples. FTIR spectroscopy was employed to analyze the spectra of CHL, GCS-IO NPs, and CHL-GCS-IO NPs at room temperature, with a resolution of 4 cm⁻¹ over the range of 4000–400 cm⁻¹. XRD was conducted on CHL, GCS-IO NPs, and CHL-GCS-IO NPs using a Bruker D2 PHASER to assess their crystalline structures. SEM with a Hitachi SU3500 was used to observe the structure and morphology of the CHL and CHL-GCS-IO NPs samples.

Additionally, x-ray photoelectron spectroscopy (XPS) using a JEOL JPS-9030 was utilized for the qualitative and quantitative surface composition analysis of CHL-GCS-IO NPs. The photothermal properties and T2-weighted magnetic resonance imaging (MRI) capabilities of the CHL-GCS-IO NPs were also evaluated to assess their multifunctionality for therapeutic and imaging applications.

### Measurement of generated oxygen

In this study, oxygen species production was detected using DHE as a molecular probe. A suspension of CHL cells (2 × 10⁷ cells/mL) and CHL-GCS-IO NPs (2 × 10⁷ cells/mL) was subjected to 660-nm laser irradiation for predetermined time intervals of 0, 5, and 10 min. After irradiation, cells were incubated with DHE (0.5 µL of a 3.15 mg/mL solution). Fluorescence changes were observed and recorded using a fluorescence microscope, and ImageJ software (National Institutes of Health, Madison, WI, USA) was used to quantify the fluorescence intensity of DHE. In parallel, an oxygen meter was employed to measure the dissolved oxygen levels in the aqueous phase, providing an additional assessment of oxygen production.

### Cytotoxicity in vitro assessment and effects of PST and PTT

This study assessed the cytotoxicity of CHL and CHL-GCS-IO NPs on MB49 bladder cancer cell lines using a standard CCK-8 assay. MB49 cells were seeded in 96-well plates and incubated at a controlled temperature for 12 h. Subsequently, different concentrations of CHL and CHL-GCS-IO NPs were added to the wells, with and without light irradiation at wavelengths of 660 and 808 nm for 10 min. For the CCK-8 assay, the CCK-8 solution was added to each well, and after 1 h of incubation, the absorbance at 450 nm was measured with a microplate reader (BioTek Instruments, Winooski, ST, USA) to determine cell viability.

In a separate experiment, MB49 cells were plated in 96-well plates at a density of 10^4^ cells per well and incubated for 24 h. Cells were then treated with CHL, GCS-IO NPs, or CHL-GCS-IO NPs for 2 h. Following treatment, cells were exposed to 10 µM of DCFH-DA (Sigma, St. Louis, MO, USA) and incubated for an additional 20 min. Afterward, the DCFH-DA solution was removed, and cells were divided into two groups: one group was kept in the dark, while the other group was irradiated with 660 nm light for 10 min. This experiment was conducted to evaluate the generation of reactive oxygen species (ROS) and PTT/PST under different conditions.

### Polarization of macrophages

Polarization of RAW264.7 macrophages was evaluated using fluorescence microscopy (Leica). RAW264.7 is a macrophage-like cell line derived from mice and obtained from ATCC. Cells were first stimulated with 100 ng/mL of lipopolysaccharide (LPS) or interleukin (IL)-4 for 24 h to respectively induce M1 or M2 polarization. Following this, cells were treated with either CHL or GCS-IO NPs, or CHL-GCS-IO NPs. After the treatment period, RAW264.7 cells were washed with a pH 7.4 buffer solution and fixed with 4% paraformaldehyde for 30 min.

Next, cells were incubated with fluorescently conjugated antibodies to assess macrophage polarization. For M2 macrophages, cells were treated with an FITC-conjugated anti-mouse CD206 (MMR) antibody (BioLegend, San Diego, CA, USA), while for M1 macrophages, Alexa Fluor^®^ 594-conjugated anti-mouse CD86 (BioLegend) was used. Incubation was carried out at 4 °C for 1 h. After staining, macrophage polarization was analyzed using either fluorescence microscopy or flow cytometry to determine the expressions of M1 and M2 markers.

### Tumor model and antitumoral efficacy tests

In this study, C57BL/6 mice, weighing approximately 20 g and aged 6 to 8 weeks, were used as the animal model for tumor experiments. Mice were obtained from BioLasco (Taipei, Taiwan), and all experimental procedures were conducted in accordance with protocols approved by the Taipei Medical University Laboratory Animal Center (approval IACUC: LAC2023-0030, SHLAC2023-0082, and LAC2023–0209). MB49 tumor-bearing C57BL/6 mice were divided into four groups: control, CHL, GCS-IO NPs, and CHL-GCS-IO NPs. When the tumor volume had reached approximately 600 mm³, each mouse in the treatment groups received an intravenous (*i.v.*) injection of either CHL, GCS-IO NPs, or CHL-GCS-IO NPs. Following the injection, mice were subjected to light and magnetic stimulation programs, consisting of a magnet application for 10 min, 660-nm laser irradiation for 10 min, or 808-nm laser irradiation for 5 min, 2 h post-injection. Tumor sizes were measured and recorded over a 14-day period using the formula: volume = (width² × length) / 2 or through ImageJ software (pixels). The volume of each injection across all groups was standardized at 100 µL.

To assess the biodistribution, pharmacokinetics, and metabolism of CHL-GCS-IO NPs, a pharmacokinetics study was performed. C57BL/6 mice (about 20–22 g) were randomly assigned to three groups: PBS, CHL, and CHL-GCS-IO NPs. Each mouse in the CHL and CHL-GCS-IO NPs groups received an i.v. injection of 100 µL containing a concentration of 10⁹ cells/mL.

### Biodistribution in vivo, performance of PTT, and fluorescence in vivo imaging

To perform in vivo biodistribution imaging, CHL and CHL-GCS-IO NPs (suspended in PBS) were administered to MB49 tumor-bearing C57BL/6 mice via an i.v. injection. The dosage was 0.15 mg/kg body weight (BW) for GCS-IO NPs and 100 µL of CHL at a concentration of 10⁹ cells/mL. Following the injection, a permanent magnet was applied to the tumor site for 10 min.

At 24 h post-injection, the tumor site was imaged using an in vivo imaging system (IVIS; Lumina III XRMS, PerkinElmer, Waltham, MA, USA). Emission wavelengths of 680 nm were selected for CHL. Results were analyzed using the IVIS software suite provided with the system. Additionally, infrared (IR) thermal imaging was conducted using an A-BF camera (MET-FLTG300 + 2, S.E.A.T Industry Technology, Kaohsiung, Taiwan) at 2-min intervals to monitor the thermal effects induced by the treatments. These imaging techniques allowed an assessment of the biodistribution and therapeutic effects of the NPs, providing insights into their potential for targeted tumor therapy.

### Histological examination

On the 14th day following the administration of the various treatments, tumor tissues were collected for histological analyses. Immunohistochemistry was performed to localize markers such as Amplex red, HIF-1α, CD8^+^ (cytotoxic T cells), CXCL12, PD-L1, CD49b (NK cells), CD11c (DC cells), CD86 (M1 macrophage polarization), CD206 (M2 macrophage polarization), and TGF-β. Additionally, hematoxylin and eosin (H&E) staining was conducted on both tumor tissues and major organs, including the heart, liver, spleen, lungs, and kidneys of treated mice.

These analyses provided insights into the immune response, polarization of macrophages, and the overall histopathology of tumors and organs, allowing an assessment of both the therapeutic efficacy and safety of the treatments.

### Statistical assessment

Quantitative results are presented as the mean values with their corresponding standard deviations (SDs). Multiple comparisons were conducted with GraphPad Prism software vers. 9.5.1 for Windows (Dotmatics, Boston, MA, USA). Statistical significance was indicated by asterisks, at * *p* < 0.05, ** *p* < 0.01, *** *p* < 0.001, and **** *p* < 0.0001.

## Results

### Formulation and characterization of GCS-IO NPs

**Fig. 2:**
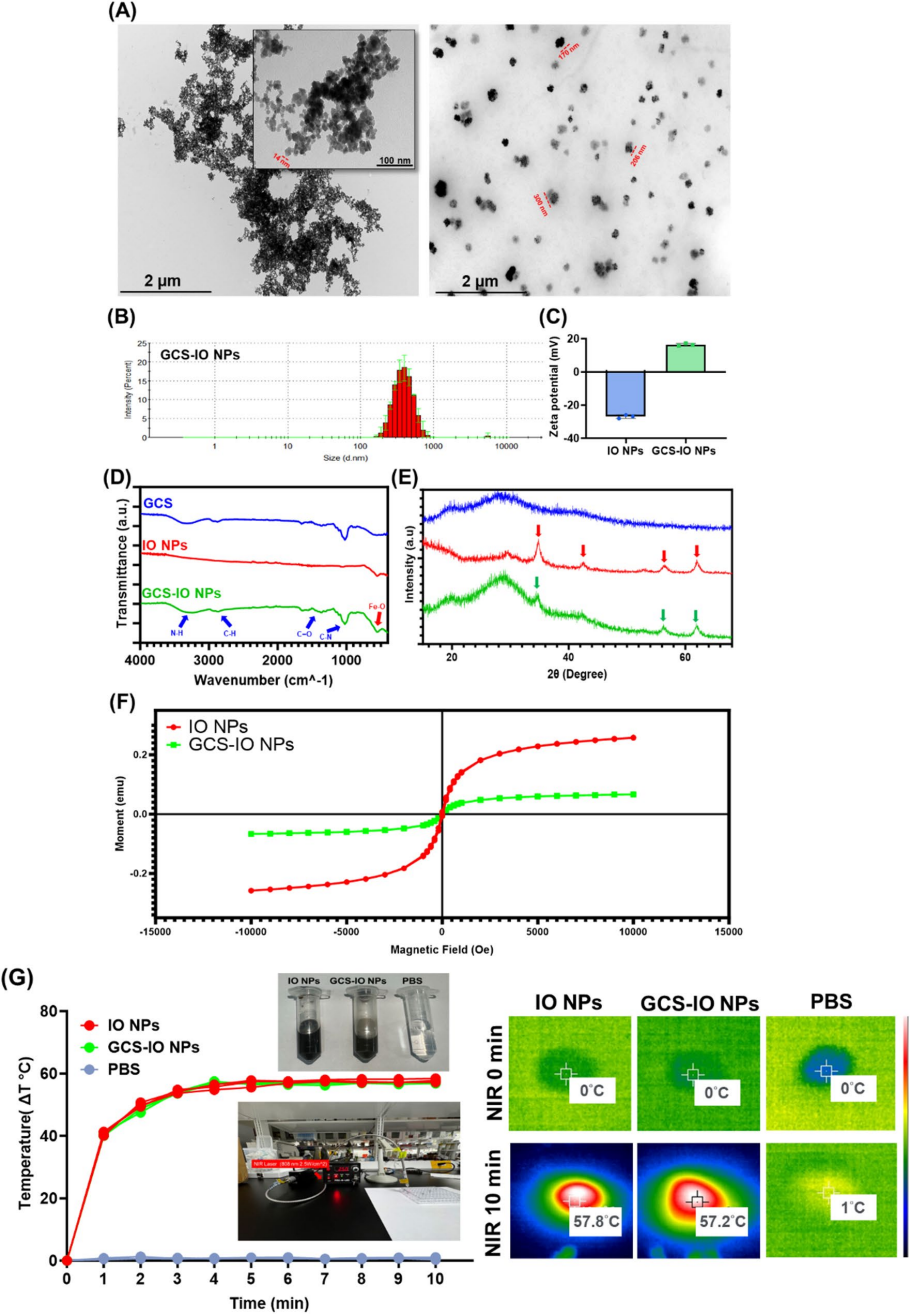
Formulation and characterization of glycol chitosan iron oxide nanoparticles (GCS-IO NPs). (**A**) Transmission electron microscopic (TEM) images showing the morphology of uncoated IO NPs (left) and GCS-IO NPs (right). GCS-IO NPs displayed a more-uniform, spherical structure with minimal aggregation, confirming successful encapsulation of IO NPs within the GCS matrix. (**B**) Particle size distribution of GCS-IO NPs measured using dynamic light scattering (DLS). GCS-IO NPs showed a mean hydrodynamic diameter of approximately below 500 nm. (**C**) Zeta potential measurements comparing IO NPs and GCS-IO NPs, indicating a significant increase in surface charge for GCS-IO NPs due to the GCS coating, contributing to improved stability. (**D**) Fourier-transform infrared (FTIR) spectra of GCS, IO NPs, and GCS-IO NPs, confirming the presence of Fe-O bonds and the successful coating of GCS around IO NPs. (**E**) X-ray diffraction (XRD) patterns of GCS, IO NPs, and GCS-IO NPs, demonstrating the crystalline structure of IO in GCS-IO NPs. (**F**) Magnetic properties of IO NPs and GCS-IO NPs measured using a superconducting quantum interference device (SQUID) magnetometer. GCS-IO NPs exhibited superparamagnetic behavior with negligible remanence and coercivity, making them suitable for magnetic targeting applications. (**G**) Photothermal performance of PBS, IO NPs, and GCS-IO NPs under 808-nm near-infrared (NIR) laser irradiation (2.45 W/cm²). GCS-IO NPs showed a significant temperature rise, confirming their photothermal conversion capability. Infrared thermal images of PBS, IO NPs, and GCS-IO NPs during NIR irradiation, illustrating the localized heating effect of GCS-IO NPs, reaching temperatures of over 50 °C, highlighting their potential for photothermal therapy (PTT)

The morphology of the GCS-IO NPs was observed using transmission electron microscopy (TEM) (Fig. 2A). Compared to uncoated IO (left), GCS-IO NPs (right) exhibited a more uniform and stable dispersion. TEM images confirmed that the IO NPs were encapsulated within the GCS matrix, forming a spherical and well-distributed structure with minimal aggregation. The GCS coating was crucial for stabilizing the particles and preventing clumping, ensuring better colloidal stability in aqueous environments [43]. The dynamic light scattering (DLS) analysis (Fig. 2B) indicated that the mean hydrodynamic diameter of GCS-IO NPs was approximately below 500 nm, a significant increase compared to bare IO NPs, highlighting the effective coating by GCS. The particle size distribution was narrow, suggesting a monodispersed system ideal for in vivo applications. Zeta potential measurements (Fig. 2C) showed that GCS-IO NPs had a higher positive charge compared to bare IO NPs, indicating enhanced colloidal stability due to surface modification with GCS, which should improve cellular interactions and prolong circulation time in the bloodstream.

The Fourier-transform infrared (FTIR) spectra of GCS-IO NPs, GCS, and IO NPs (Fig. 2D) confirmed the successful encapsulation of IO NPs within the GCS matrix. Characteristic peaks at 3200–3500 cm⁻¹ corresponding to O-H and N-H stretching vibrations [44] in GCS-IO NPs were observed, confirming the presence of hydroxyl and amine groups from GCS. Peaks at around 600 cm⁻¹, attributed to Fe-O bonds, were clearly visible in IO NPs and GCS-IO NPs, further verifying the presence of IO within the NP system. The x-ray diffraction (XRD) analysis (Fig. 2E) revealed distinct diffraction peaks at 30°, 35°, 43°, and 57°, which corresponded to the crystalline structure of IO NPs [45]. A broadening of peaks in GCS-IO NPs indicated that the GCS coating introduced some amorphous character, yet the overall crystalline structure of the IO core remained intact, supporting its use for magnetic applications. Magnetic characterization using a superconducting quantum interference device (SQUID) magnetometer (Fig. 2F) demonstrated that GCS-IO NPs exhibited superparamagnetic behavior, with a near-zero remanence and coercivity. This is a desirable trait for biomedical applications such as magnetic resonance imaging (MRI) and targeted drug delivery, as the NPs can be efficiently guided using an external magnetic field without retaining residual magnetization once the field is removed. The reduced magnetic moment compared to bare IO NPs was attributed to the GCS coating, which slightly hindered the magnetic response, but not to a degree that would impair its utility in magnetic-targeted applications. The photothermal conversion efficiency of GCS-IO NPs was evaluated under 808-nm near-infrared (NIR) laser irradiation (2.45 W/cm²) in an aqueous dispersion (Fig. 1G). Compared to phosphate-buffered saline (PBS) and IO NPs, GCS-IO NPs exhibited a significantly higher temperature increase, reaching over 50 °C after 10 min of irradiation. This superior photothermal performance can be attributed to the combination of IO’s intrinsic photothermal properties [46] and enhanced colloidal stability provided by the GCS coating. The data suggested that GCS-IO NPs can be highly effective for photothermal therapy (PTT) applications, allowing for localized hyperthermia in tumor tissues with minimal damage to surrounding healthy tissues. Infrared thermal imaging (Fig. 1G) further confirmed the photothermal effect, showing a stark contrast in temperature changes between the PBS control and GCS-IO NPs group. Images highlighted the localized heating effect of GCS-IO NPs upon laser irradiation, making them promising candidates for remote non-invasive cancer therapies that leverage hyperthermia.

Results presented in Fig. 1 collectively demonstrate the successful formulation and characterization of GCS-IO NPs, which combine the magnetic properties of IO with the biocompatibility and stability provided by the GCS coating. The NPs exhibited an excellent photothermal conversion efficiency, magnetic responsiveness, and stable dispersion, making them well-suited for multifunctional theranostic applications, including MRI, PTT, and targeted drug delivery.

### Formulation and characterization of CHL-GCS-IO NPs

**Fig. 3:**
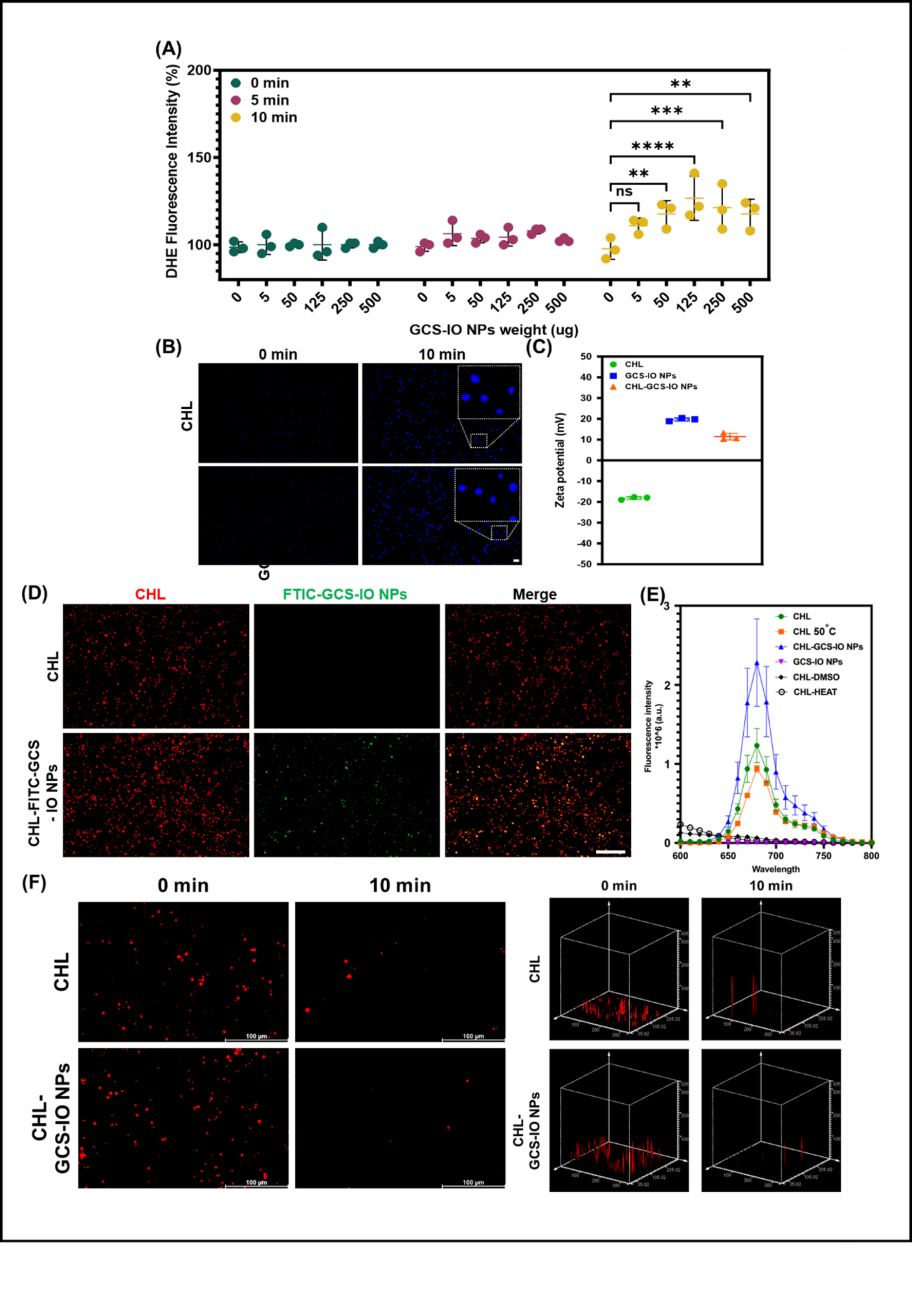
Characterization and performance of *Chlorella* glycol chitosan iron oxide nanoparticles (CHL-GCS-IO NPs). (**A**) DHE fluorescence intensity data demonstrating oxygen generation by CHL (10^7^ cells/mL) formulated with various concentrations of GCS-IO NPs (0, 5, 50, 125, 250, and 500 µg) under 660-nm laser irradiation for 0, 5, and 10 min. Data show a time- and concentration-dependent increase in oxygen production, with high reactive oxygen species (ROS) generation tendency at higher NP concentrations and longer irradiation times. (**B**) Fluorescence microscopic images showing DHE fluorescence in CHL and CHL-GCS-IO NPs before and after 10 min of 660-nm laser irradiation. CHL-GCS-IO NPs exhibited higher fluorescence intensities, indicating enhanced oxygen generation. Scale bar: 10 μm. (**C**) Zeta potential measurements for CHL, GCS-IO NPs, and CHL-GCS-IO NPs. The shift in the zeta potential from − 20 mV for CHL to + 10 mV for CHL-GCS-IO NPs suggests successful conjugation of GCS-IO NPs and improved colloidal stability. (**D**) Fluorescence microscopy showing conjugation of GCS-IO NPs (green FITC fluorescence) onto CHL (red chlorophyll fluorescence). The merged image confirms successful conjugation, with distinct fluorescent signals for FITC and chlorophyll, indicating that the photosynthetic and magnetic properties were retained. Scale bar: 50 μm. (**E**) Comparison of chlorophyll activities across various formulations (CHL, CHL-GCS-IO NPs, GCS-IO NPs, CHL-DMSO, and CHL-HEAT). CHL-GCS-IO NPs demonstrated slightly increased chlorophyll activity compared to untreated CHL. (**F**) RDPP data of CHL and CHL-GCS-IO NPs evaluated at 0 min and 10 min light irradiation

The dihydroethidium (DHE) fluorescence intensity data (Fig. 3A) revealed the ability of CHL (10^7^ cells/mL) plus various amounts of GCS-IO NPs to produce reactive oxygen upon light irradiation, evaluated for various GCS-IO NPs feeding amounts (0, 5, 50, 125, 250, and 500 µg). The DHE assay was used to detect oxygen generation [47] under 660-nm laser irradiation for 0, 5, and 10 min. The formulated CHL plus GCS-IO NPs exhibited a time- and concentration-dependent increase in oxygen generation, as indicated by the significant rise in the DHE fluorescence intensity at higher NP feeding amounts (250 and 500 µg) and an extended exposure time (10 min). The data suggested that this oxygen could efficiently modulate oxygen levels within the TME, which is critical for overcoming hypoxic conditions typically found in solid tumors [48]. Specifically, 10 min of irradiation at a 125-µg feed amount resulted in a nearly 25% increase in the fluorescence intensity, indicating robust oxygen production compared to the other groups, all of which showed minimal oxygen generation. Considering the highest efficacy, the notable enhancement in oxygenation supported selecting CHL-GCS-IO NPs at a concentration of 125 µg for subsequent experiments. This dosage proved to be an effective therapeutic platform for alleviating tumor hypoxia and enhancing the efficacy of dynamic therapies, such as PST. Figure 3B further supports these findings with fluorescence microscopic images. The difference in the fluorescence intensity before and after 10 min of 660-nm light irradiation demonstrated that the formulated CHL-GCS-IO NPs exhibited significantly higher oxygen production compared to untreated CHL. The pronounced increase in DHE fluorescence (blue signal) after 10 min of exposure indicated effective ROS generation in the presence of NPs. The morphological uniformity of the fluorescence distribution also highlighted the efficient interaction between light irradiation and CHL-GCS-IO NPs, resulting in sustained oxygen release. These results suggested the potential of this system to overcome hypoxia-driven chemoresistance by modulating the TME. A zeta potential analysis (Fig. 3C) was performed to assess the colloidal stability [49] of CHL, GCS-IO NPs, and CHL-GCS-IO NPs. Bare CHL exhibited a negative zeta potential of approximately -20 mV, typical for microalgae due to the presence of negatively charged functional groups on their surface. GCS-IO NPs showed a positive zeta potential (ca. +20 mV), attributed to the cationic nature of GCS used for coating the IO NPs. Upon conjugation of the GCS-IO NPs to CHL cells, the zeta potential of CHL-GCS-IO NPs shifted slightly to around + 10 mV, suggesting GCS-IO NPs attachment and charge neutralization between the anionic CHL surface and cationic GCS-IO NPs. The positively charged surface of CHL-GCS-IO NPs enhanced their stability, improving cellular interactions and reducing aggregation, which is crucial for in vivo applications, especially for prolonging circulation times and effective tumor targeting. The zeta potential measurements of our developed formulation at different time points (0 days, 1 day, and 7 days) in PBS. The results show that the zeta potential remains consistently above + 10 mV across all time points, indicating good stability of the CHL-GCS-IO NPs in PBS over time (Figure S1A). Fluorescence microscopic data (Fig. 3D) indicated the conjugation of GCS-IO NPs (with green FITC fluorescence) onto CHL (red chlorophyll fluorescence). In CHL-GCS-IO NPs samples, a distinct overlay of FITC (green) and chlorophyll (red) fluorescence was observed, confirming the formation of hybrid CHL-GCS-IO NPs. Merged images revealed a homogeneous distribution of the GCS-IO NPs on the CHL surface, signifying efficient conjugation between GCS-IO NPs and CHL cells. Fluorescent signals from both FITC and chlorophyll remained distinct and stable after conjugation, demonstrating that the conjugation process did not adversely affect the inherent properties of either the GCS-IO NPs or CHL. These results suggest that CHL-GCS-IO NPs retained photosynthetic capabilities of CHL while integrating magnetic and photothermal properties of GCS-IO NPs, creating a multifunctional system for theranostic applications. Figure 3E compares chlorophyll activities of different formulations, showing the fluorescence intensity of chlorophyll [50] in CHL, CHL-GCS-IO NPs, GCS-IO NPs, CHL-DMSO, and CHL-HEAT under various conditions. CHL-GCS-IO NPs exhibited slightly increased chlorophyll activity compared to untreated CHL alone, which is expected due to the positive attenuation caused by NP binding. However, the fluorescence intensity of CHL-GCS-IO NPs remained significantly higher than those of the CHL-DMSO and CHL-HEAT groups, indicating that chlorophyll activity was preserved despite conjugation with GCS-IO NPs. The CHL sample exposed to 50 °C retained a fluorescence intensity of about 1 unit with an emission wavelength between 650 and 700 nm, indicating stable optical properties under thermal conditions.

This preserved chlorophyll activity is critical for the functionality of CHL as a photosynthetic agent, enabling it to continuously produce oxygen under light irradiation, which is essential for enhancing PST therapeutic effects in tumor environments. In Fig. 3F, the photobleaching behavior and fluorescence stability of CHL and CHL-GCS-IO NPs were assessed using the RDPP assay to evaluate oxygen production at 0 and 10 min of light irradiation. At 0 min, both CHL and CHL-GCS-IO NPs displayed strong fluorescence signals, indicating high initial fluorescence intensity. However, after 10 min of irradiation, CHL exhibited a faint red fluorescence, while CHL-GCS-IO NPs showed less detectable fluorescence. This suggests that CHL underwent partial photobleaching, retaining residual fluorescence, whereas CHL-GCS-IO NPs facilitated complete fluorescence quenching. The observed difference in fluorescence retention may be attributed to the interaction between CHL and the nanoparticle matrix, which likely enhances photobleaching or promotes signal quenching. These results provide insights into the oxygen-generating capacity of CHL-GCS-IO NPs in comparison to free CHL.

**Fig. 4:**
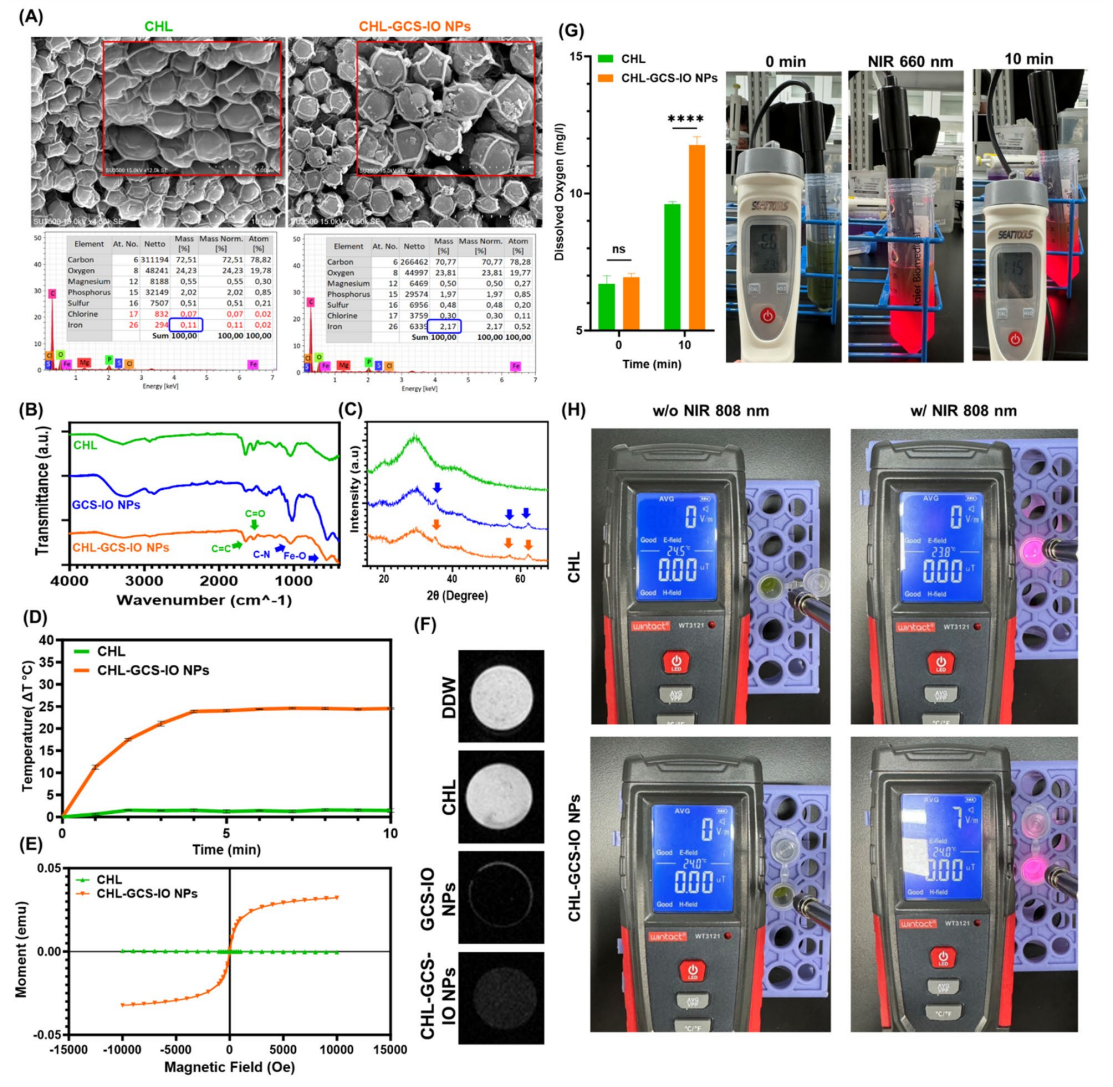
Characterization of *Chlorella* glycol chitosan iron oxide nanoparticles (CHL-GCS-IO NPs) (**A**) Scanning electron microscopic (SEM)-energy-dispersive x-ray spectroscopic (EDS) analysis. SEM images comparing surface morphologies of CHL and CHL-GCS-IO NPs. The SEM image of CHL shows a smoother surface, while CHL-GCS-IO NPs exhibit surface roughness, indicative of successful conjugation with GCS-IO NPs. Elemental analysis by EDS demonstrates a significant increase in iron (Fe) content in CHL-GCS-IO NPs (2.17%) compared to CHL (0.11%), confirming NP attachment. (**B**) Fourier-transform infrared (FTIR) spectroscopy: FTIR spectra of CHL, GCS-IO NPs, and CHL-GCS-IO NPs. The spectrum of CHL-GCS-IO NPs shows characteristic peaks of C = O stretching (~ 1700 cm⁻¹), C-N stretching (~ 1400 cm⁻¹), and Fe-O (~ 600 cm⁻¹), confirming successful conjugation and preservation of bioactivity. (**C**) X-ray diffraction (XRD) analysis: XRD patterns of CHL, GCS-IO NPs, and CHL-GCS-IO NPs. Crystalline peaks corresponding to Fe₃O₄ (iron oxide) were observed in CHL-GCS-IO NPs, confirming the incorporation of magnetic NPs into the *Chlorella* system. (**D**) Photothermal heating effect (photothermal therapy (PTT)): Temperature elevation profiles of CHL and CHL-GCS-IO NPs under 808-nm NIR irradiation. CHL-GCS-IO NPs exhibited a substantial temperature increase of over 20 °C after 10 min, highlighting their potential for PTT, whereas CHL alone showed a minimal temperature change. (**E**) Superconducting quantum interference device (SQUID) magnetization curves: SQUID analysis of CHL and CHL-GCS-IO NPs. The magnetization curve for CHL-GCS-IO NPs indicates superparamagnetic behavior with significantly higher saturation magnetization (Ms), essential for magnetic guidance and in vivo therapeutic applications. (**F**) T2-weighted magnetic resonance imaging (MRI) analysis: T2-weighted MRI images of double-distilled water (DDW), CHL, GCS-IO NPs, and CHL-GCS-IO NPs. CHL-GCS-IO NPs demonstrated strong T2 contrast, enhancing their potential as MRI contrast agents for precise tumor imaging in theranostic applications. (**G**) Photosynthetic oxygen production under light irradiation. The graph compares the oxygen production rates of *Chlorella* (CHL) and *Chlorella*-conjugated glycol iron oxide nanoparticles (CHL-GCS-IO NPs) under identical light exposure conditions. CHL-GCS-IO NPs exhibit significantly enhanced oxygen production compared to CHL, indicating improved photosynthetic efficiency due to the presence of glycol iron oxide nanoparticles. (**H**) Electromagnetic wave responses of *Chlorella* (CHL) and CHL-GCS-IO NPs under light irradiation. The electromagnetic field (E-field) measurements demonstrate a negligible change in CHL samples under light exposure, while CHL-GCS-IO NPs exhibit a substantial increase in electromagnetic activity, likely attributed to the conductive and electromagnetic-responsive properties of the glycol iron oxide nanoparticles

SEM images and elemental composition data from energy-dispersive X-ray spectroscopy (EDS) provided insights into structural differences between CHL and CHL-GCS-IO NPs (Fig. 4A). SEM images revealed clear distinctions in morphology, with CHL-GCS-IO NPs showing surface roughness and enhanced structural definition compared to the smoother surface of CHL alone. The iron (Fe) content significantly increased in CHL-GCS-IO NPs, confirming the successful conjugation of IO NPs to *Chlorella* cells, with the Fe mass increasing from 0.11% in CHL to 2.17% in CHL-GCS-IO NPs. This substantial rise in the Fe content is key to the enhanced magnetic properties of the system, enabling magnetic guidance and potential for theranostic applications. Additionally, other elemental components, such as oxygen and sulfur, remained relatively constant, indicating that the core structure of *Chlorella* was preserved during the NP attachment process.

FTIR spectroscopy (Fig. 4B) was used to identify functional groups present in GCS-IO NPs, CHL, and CHL-GCS-IO NPs. For GCS-IO NPs, characteristic absorption bands were observed for GCS and IO, including peaks corresponding to Fe-O and C-N bonds. In CHL-GCS-IO NPs, the C = O stretching peak (~ 1700 cm⁻¹) [51] and the C-N stretching at ~ 1000 cm⁻¹ [52] were indicative of successful conjugation between the NPs and the *Chlorella* surface. The presence of the Fe-O absorption band (at ~ 600 cm⁻¹) [53] confirmed the attachment of IO NPs. The overlap of these peaks suggested that the GCS-IO NPs did not interfere with the key functional groups of CHL, preserving the system’s bioactivity while enhancing its functional properties.

The XRD analysis (Fig. 4C) revealed the crystalline structure of CHL-GCS-IO NPs compared to CHL and GCS-IO NPs. Distinct peaks corresponding to crystalline phases of IO were observed in both GCS-IO NPs and CHL-GCS-IO NPs, confirming the presence of magnetite/maghemite NPs. Diffraction peaks at around 2θ = 30° and 60° well matched with characteristic peaks of Fe₃O₄, further validating the successful incorporation of magnetic NPs into the CHL system. Notably, CHL alone displayed a relatively amorphous pattern, while CHL-GCS-IO NPs showed additional crystalline peaks due to IO, indicating that the NPs contributed to the overall crystalline structure without disrupting CHL’s natural morphology.

The photothermal heating effect of CHL-GCS-IO NPs was examined under 808-nm NIR irradiation (Fig. 4D). CHL-GCS-IO NPs exhibited a significant temperature change increase compared to CHL alone, with temperatures rising by over 20 °C after 10 min of irradiation. This demonstrated the potential of CHL-GCS-IO NPs to effectively generate heat, supporting their use in PTT applications. In contrast, the temperature of CHL alone remained nearly constant, indicating that CHL exhibited no intrinsic photothermal properties without the attached IO NPs. The rapid and substantial temperature rise in the CHL-GCS-IO NPs group was crucial for inducing hyperthermia in tumor tissues, which can contribute to the destruction of cancer cells.

A SQUID analysis (Fig. 4E) was performed to evaluate the magnetic properties of CHL-GCS-IO NPs compared to CHL alone. The magnetization curve for CHL-GCS-IO NPs showed clear superparamagnetic behavior, with a significant increase in the magnetic moment compared to the non-magnetic CHL control. The saturation magnetization (Ms) of CHL-GCS-IO NPs was much higher, further confirming the successful incorporation of IO NPs [54]. This magnetic responsiveness is critical for the guidance and actuation of CHL-GCS-IO NPs in vivo under external magnetic fields, facilitating targeted delivery to tumor sites and enhancing therapeutic outcomes.

T2-weighted MRI results (Fig. 4F) demonstrated the potential of CHL-GCS-IO NPs as contrast agents. While CHL and double-distilled water (DDW) showed minimal contrast in MRI images, GCS-IO NPs and CHL-GCS-IO NPs produced strong T2 contrast, as indicated by the darker signal in MRI scans. The CHL-GCS-IO NPs group exhibited superior T2-weighted contrast compared to GCS-IO NPs alone, likely due to the enhanced particle dispersion and stability provided by attachment to CHL. These results suggested that CHL-GCS-IO NPs hold promise as MRI contrast agents for non-invasive tumor imaging, enabling precise localization of tumors in addition to their therapeutic functions. CHL-GCS-IO NPs characterization indicated their multifunctional properties, combining the photosynthetic activity of CHL with the magnetic and photothermal properties of GCS-IO NPs. These attributes make CHL-GCS-IO NPs an ideal platform for multimodal theranostic applications, capable of MRI-guided therapy, photothermal ablation, and immune activation through photosynthetic oxygen generation. The robust magnetic properties, high photothermal conversion efficiency, and excellent MRI contrast further support their use in dynamic cancer therapies, targeting solid tumors in hypoxic microenvironments.

Figure 4G and H present the results of photosynthetic oxygen evolution and the electromagnetic wave responses under light irradiation for *Chlorella* (CHL) cells and *Chlorella*-conjugated glycol iron oxide nanoparticles (CHL-GCS-IO NPs). The photosynthetic oxygen production data in Fig. 4G demonstrate a significant enhancement in oxygen evolution upon light irradiation for both CHL and CHL-GCS-IO NPs, with CHL-GCS-IO NPs showing a markedly higher output. This suggests that the conjugation with glycol iron oxide nanoparticles enhances the efficiency of photosynthesis, likely due to the improved light-harvesting capability of the nanoparticle-functionalized cells. The CHL at 25 °C and 50 °C showed photosynthetic oxygen production even at elevated temperature (Figure S1B). Previous studies have indicated that microalgae may exhibit some level of tolerance to both thermal stress and free radicals [55–57]. It is important to highlight that the 808 nm light used for photothermal therapy (PTT) is applied in the final stage of the treatment. Prior to this, CHL-GCS-IO NPs are exposed to 660 nm light, which is responsible for driving the photosynthetic processes. In the final step, the CHL-GCS-PPy NPs undergo exposure to 808 nm light for the PTT treatment. While this step may potentially cause the death of the microalgae, any surviving CHL-GCS-PPy NPs would be eliminated by the body’s immune system and subsequently excreted.

In Fig. 4H, the electromagnetic wave data reveal distinct responses between the CHL and CHL-GCS-IO NPs samples. The electromagnetic field (E-field) measurements under light irradiation show negligible changes for pure CHL, while CHL-GCS-IO NPs exhibit a noticeable increase in electromagnetic activity. This enhanced response in CHL-GCS-IO NPs can be likelyattributed to the conductive properties of the glycol iron oxide nanoparticles, which facilitate the generation and transmission of electromagnetic signals under illumination.

These findings highlight the dual functionality of CHL-GCS-IO NPs, combining enhanced photosynthetic activity with electromagnetic responsiveness. The integration of nanoparticles with *Chlorella* cells not only amplifies biological photosynthesis but also introduces a potential for applications in biohybrid systems, such as light-driven electromagnetic devices or bioenergy systems.

### In vitro biochemical assays

**Fig. 5:**
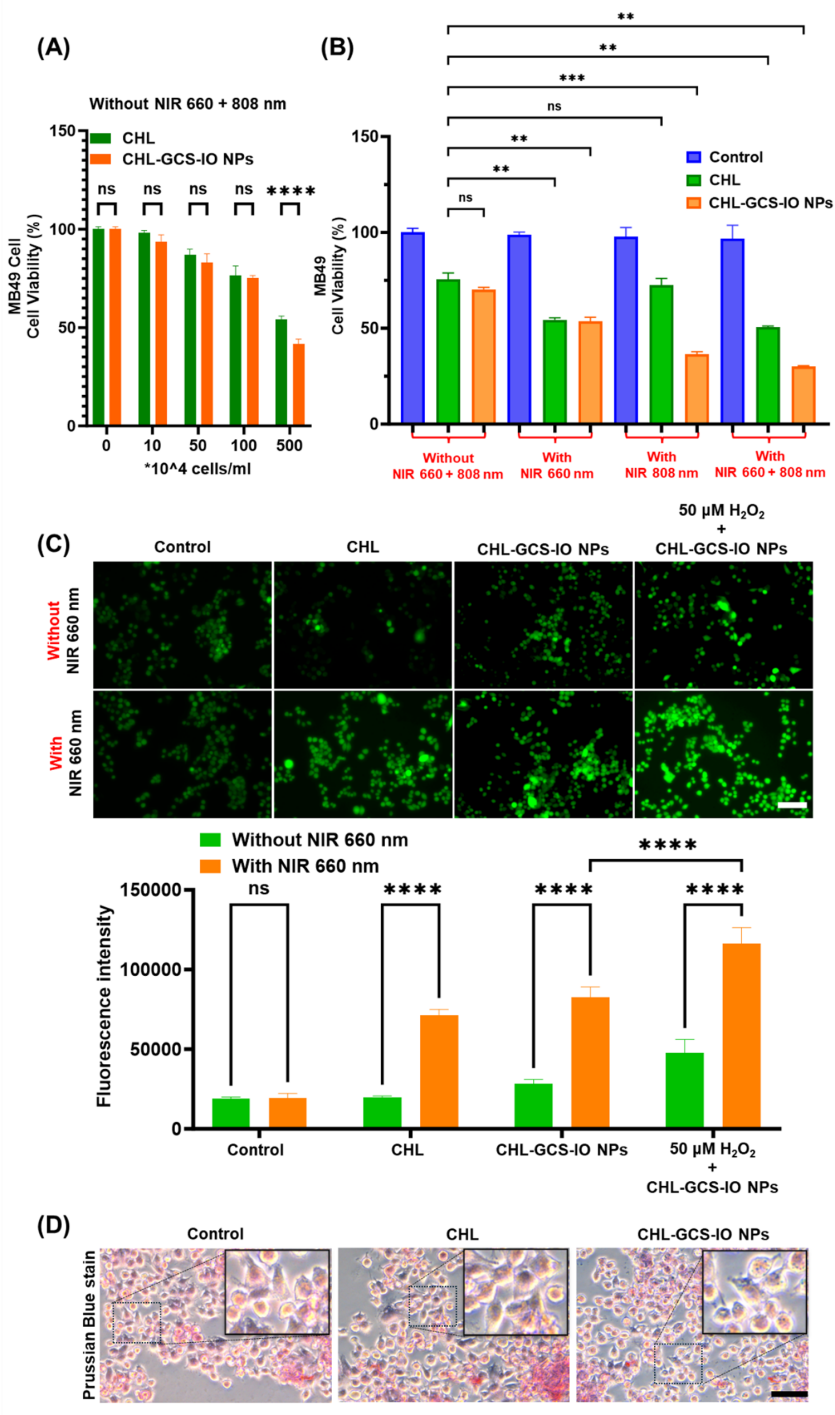
Evaluation of MB49 cell viability and reactive oxygen species (ROS) generation following treatment with *Chlorella* (CHL) and CHL-glycol chitosan iron oxide nanoparticles (CHL-GCS-IO NPs) under near infrared (NIR) irradiation. (**A**) Cell viability of MB49 cells treated with CHL and CHL-GCS-IO NPs at various concentrations (0–500 µg/mL) without NIR irradiation. Both CHL and CHL-GCS-IO NPs exhibited moderate cytotoxicity, with cell viability remaining above 60% (0–100 µg/mL). Statistical significance: ns, not significant, **** *p* < 0.0001. (**B**) Cell viability of MB49 cells treated with CHL and CHL-GCS-IO NPs with or without NIR irradiation (660 + 808 nm). CHL-GCS-IO NPs, when irradiated with 660- and 808 nm-light, resulted in a significant decrease in cell viability (~ 70% cell death). In contrast, CHL alone produced a minimal reduction in viability. (**C**) ROS generation by MB49 cells, as determined by a DCFH-DA assay. Fluorescence microscopic images (top: without NIR; bottom: with 660-nm NIR) and corresponding quantitative analysis (bottom graph) demonstrating significant ROS production induced by CHL and CHL-GCS-IO NPs under NIR irradiation. The CHL-GCS-IO NPs group exhibited significantly higher ROS generation than CHL alone, and the addition of H₂O₂ further amplified ROS production. Scale bar: 100 μm. (**D**) Cellular Prussian blue staining. Scale bar: 50 μm

The cell viability assay presented in Fig. 5A highlights the effects of CHL and CHL-GCS-IO NPs on MB49 bladder cancer cells in the absence of NIR light irradiation. Without NIR activation, CHL and CHL-GCS-IO NPs moderately reduce MB49 cell viability at concentrations of up to 100 × 10^4^ cells/mL, with both treatment groups maintaining over 60% viability across all concentrations. This indicates that without PTT or PST activation, CHL and CHL-GCS-IO NPs exhibited moderate cytotoxicity in the absence of light stimulation. Importantly, CHL-GCS-IO NPs showed slightly lower cell viability at the high concentration (100 × 10^4^ cells /mL), but the difference was not statistically significant (ns), further confirming that NP conjugation did not inherently induce cytotoxicity under non-irradiative conditions.

In contrast, Fig. 5B demonstrates the combined effects of CHL, CHL-GCS-IO NPs, and NIR irradiation (660 and 808 nm) on MB49 cell viability. Being subjected to NIR irradiation, cell viability was significantly reduced by CHL-GCS-IO NPs in a wavelength-dependent manner. Under 660-nm irradiation, CHL-GCS-IO NPs caused a substantial decrease in cell viability (approximately 50% cell death), whereas 808-nm irradiation enhanced this effect, leading to an even more-pronounced reduction in viability (~ 65% cell death). The combination of both 660- and 808-nm wavelengths resulted in the highest level of cell death (~ 70%), underscoring the synergistic effects of PTT and PST mediated by CHL-GCS-IO NPs. In comparison, CHL alone also induced some reduction in cell viability under NIR irradiation, but its effects were significantly less pronounced than CHL-GCS-IO NPs. This data confirmed the superior phototherapeutic efficacy of CHL-GCS-IO NPs, particularly when both wavelengths were employed, likely due to enhanced oxygen generation and heat production facilitated by IO NPs.

Results of in vitro qualitative and quantitative analyses (Fig. 5C) indicated cellular ROS generation using the DCFH-DA assay under NIR 660-nm irradiation. Fluorescence images and intensity quantification showed a clear difference in ROS production across the various treatment groups. In the absence of NIR irradiation, all groups, including the control, CHL, and CHL-GCS-IO NPs groups, exhibited minimal ROS generation, as indicated by the weak green fluorescence. However, upon 660-nm NIR irradiation, both CHL and CHL-GCS-IO NPs induced substantial ROS generation, with CHL-GCS-IO NPs exhibiting a significantly stronger fluorescence signal (*p* < 0.0001), indicating higher ROS levels compared to CHL alone. The inclusion of H_2_O_2_ in the CHL-GCS-IO NPs group further amplified ROS production, demonstrating the ability of the formulation to catalyze H_2_O_2_ into oxygen and enhance the oxygen-generating effect (*p* < 0.0001). Quantitative data, as measured by ImageJ software, confirmed these findings, with CHL-GCS-IO NPs producing the highest fluorescence intensity, reinforcing their capability for potent ROS-mediated cancer cell destruction through PST.

Figure 5D shows that CHL-GCS-IO NPs significantly induce iron accumulation in MB49 cells, as evidenced by Prussian blue staining, compared to control and CHL-only treatments. This effective delivery of iron oxide nanoparticles through Chlorella likely initiates ferroptosis, a form of programmed cell death linked to lipid peroxidation. The reduction in MB49 cell viability in the CHL-GCS-IO NP group supports this therapeutic effect. Consequently, these bioengineered nanoparticles demonstrate a dual function, combining Chlorella’s biocompatibility with the cytotoxic potential of iron-induced ferroptosis, presenting a promising cancer therapy strategy.

Together, these results underscore the dual PTT and PST capabilities of CHL-GCS-IO NPs, making them a highly effective theranostic platform for bladder cancer therapy, particularly in overcoming hypoxia-driven resistance within the TME. Their ability to generate substantial ROS and induce significant cell death under NIR irradiation highlights their potential for dynamic cancer treatment applications. The combination of IO NPs and CHL enhanced both magnetic responsiveness and photosynthetic oxygen production, offering a multifaceted approach to tumor therapy that integrates PTT, PST, and immune-activating strategies.

**Fig. 6:**
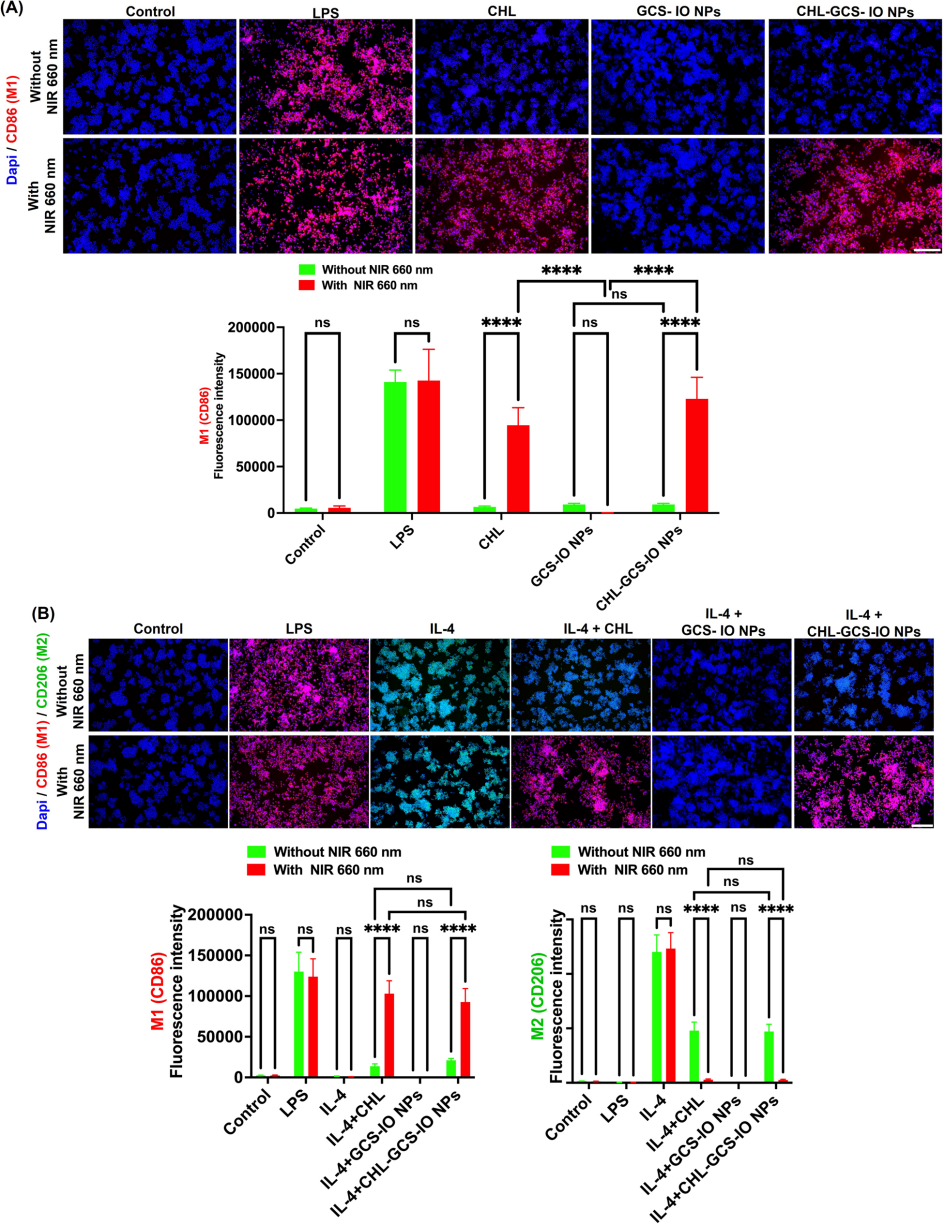
Polarization of RAW264.7 macrophages induced by *Chlorella* (CHL) and CHL-glycol chitosan iron oxide nanoparticles (GCS-IO NPs) with and without near infrared (NIR) irradiation. (**A**) M0 to M1 polarization (CD86 expression). Fluorescence microscopic images of RAW264.7 macrophages showing CD86 (M1 marker, red) and nuclei stained with DAPI (blue) under various treatments: control, lipopolysaccharide (LPS), CHL, GCS-IO and CHL-GCS-IO NPs, with and without 660-nm NIR irradiation. LPS treatment showed significant CD86 expression, indicative of M1 polarization. The 660-nm NIR irradiation of CHL-GCS-IO NPs significantly enhanced M1 polarization, as indicated by an increased CD86 fluorescence intensity compared to CHL treatment alone. Quantitative analysis of the fluorescence intensity, using ImageJ software, confirmed the significant increase in CD86 expression by CHL-GCS-IO NPs under NIR irradiation. (**B**) M2 to M1 re-polarization (CD206 to CD86 expression shift). Fluorescence microscopic images showing CD206 (M2 marker, green) and CD86 (M1 marker, red) expressions by RAW264.7 macrophages treated with IL-4 (an M2 polarization inducer), followed by CHL and CHL-GCS-IO NPs, with and without 660-nm NIR irradiation. Without NIR, IL-4 maintained high CD206 and low CD86 expression, indicating strong M2 polarization. With NIR irradiation, CHL-GCS-IO NPs induced a significant shift, reducing CD206 and enhancing CD86 expression, indicating re-polarization from M2 to M1. Quantitative analysis using ImageJ software demonstrated a significant increase in CD86 and a decrease in CD206 expression after NIR irradiation in the CHL-GCS-IO NP group (**** *p* < 0.0001). Scale bar: 200 μm

Fluorescence microscopic results in Fig. 6A highlight polarization of RAW264.7 macrophages from the M0 state to the M1 state, marked by cluster of differentiation 86 (CD86; M1) expression, upon treatment with CHL and CHL-GCS-IO NPs. Without NIR irradiation, the lipopolysaccharide (LPS)-treated group showed high CD86 expression, indicating efficient polarization into M1 macrophages. In contrast, treatment with CHL, and CHL-GCS-IO NPs produced minimal CD86 expression under these conditions, suggesting a limited ability to induce M1 polarization in the absence of external stimulation. The GCS-IO NPs produced less CD86 expression with and without NIR irradiation. Upon 660-nm NIR irradiation, CHL-GCS-IO NPs produced significantly increased CD86 expression, indicating enhanced M1 polarization compared to CHL alone. This effect highlights the potential of CHL-GCS-IO NPs to modulate macrophage polarization, driving proinflammatory (M1) activation under light stimulation. Qualitative data from microscopic images were supported by a quantitative analysis using ImageJ software, showing a significant increase in fluorescence intensities for the CHL-GCS-IO NPs group after NIR irradiation, further confirming the role of NIR-activated CHL-GCS-IO NPs in promoting M1 polarization. The ability to modulate macrophage activation could play a crucial role in reshaping the tumor immune microenvironment, making it more conducive to immune-mediated cancer cell clearance.

Figure 6B presents data on the re-polarization of M2 macrophages (induced by interleukin (IL)-4) back to the M1 state using CHL and CHL-GCS-IO NPs, marked by both CD206 (M2) and CD86 (M1) expressions. Without NIR irradiation, IL-4-treated macrophages displayed high CD206 (M2) expression, indicative of successful M2 polarization. The addition of CHL or CHL-GCS-IO NPs had partial effects on reducing M2 polarization under non-irradiated conditions. The GCS-IO NPs produced no CD206 expression with and without NIR irradiation. However, with NIR irradiation, CHL-GCS-IO NPs caused significantly increased CD86 expression (*p* < 0.0001), while simultaneously reducing CD206 expression, indicating an effective re-polarization from the anti-inflammatory M2 phenotype (mimicking the TME) back to the proinflammatory M1 state. CHL treatment alone also demonstrated some ability to re-polarize macrophages from M2 to M1, although to a lesser extent than by CHL-GCS-IO NPs. These results suggested that light-triggered activity of CHL-GCS-IO NPs can effectively switch macrophages from an immunosuppressive to a proinflammatory phenotype, which is critical for enhancing antitumor immune responses. The quantitative analysis using ImageJ software supported these findings, showing a significant increase in M1 markers and a decrease in M2 markers after NIR irradiation in the CHL-GCS-IO NPs group (*p* < 0.0001). This re-polarization ability represents a promising strategy for overcoming tumor-associated macrophage (TAM)-mediated immunosuppression in the TME. The immunofluorescence data suggested that immune cells, such as macrophages, can be educated, trained, and reprogrammed in response to PST with CHL-GCS-IO NPs upon 660-nm irradiation. Together, data from Fig. 6 underscore the dual capability of CHL-GCS-IO NPs to drive macrophage polarization toward a tumor-suppressive M1 phenotype, both from naïve macrophages and re-polarized M2 macrophages. The NIR-triggered modulation of macrophage activity suggests a novel immunotherapeutic approach that could be integrated with other cancer treatment modalities to enhance the immune response within the TME, potentially leading to improved therapeutic outcomes.

The blood test analysis (Figure S1C) provides a comparative evaluation of blood cell populations over time (14 days) across different formulation-treated groups, with a focus on red blood cells (RBCs; reference range: approximately 7.8–10.6 M/µL), reticulocytes (reference range: approximately 200–500 K/µL), and white blood cells (WBCs; reference range: approximately 2–10 K/µL).

In the control group, the RBC count was 10.47 M/µL, reticulocyte count was 476.4 K/µL, and WBC count was 5.04 K/µL. By day 14, the CHL-GCS-IO NPs (mag.) group showed a slight recovery in RBCs to 8.67 M/µL, a mild reduction in reticulocytes to 512.5 K/µL, and a stable WBC count at 6.34 K/µL.

These results suggest that treatment with CHL-GCS-IO NPs does not significantly affect hematopoiesis and maintains blood cell levels within biosafe reference ranges over time. Distinct blood cell types are represented by specific colors in the scatter plots—RBCs (red), reticulocytes (purple), and WBCs (cyan)—which highlight the distribution and clustering patterns of these populations across both control and treated groups.

### In vivo examinations

**Fig. 7:**
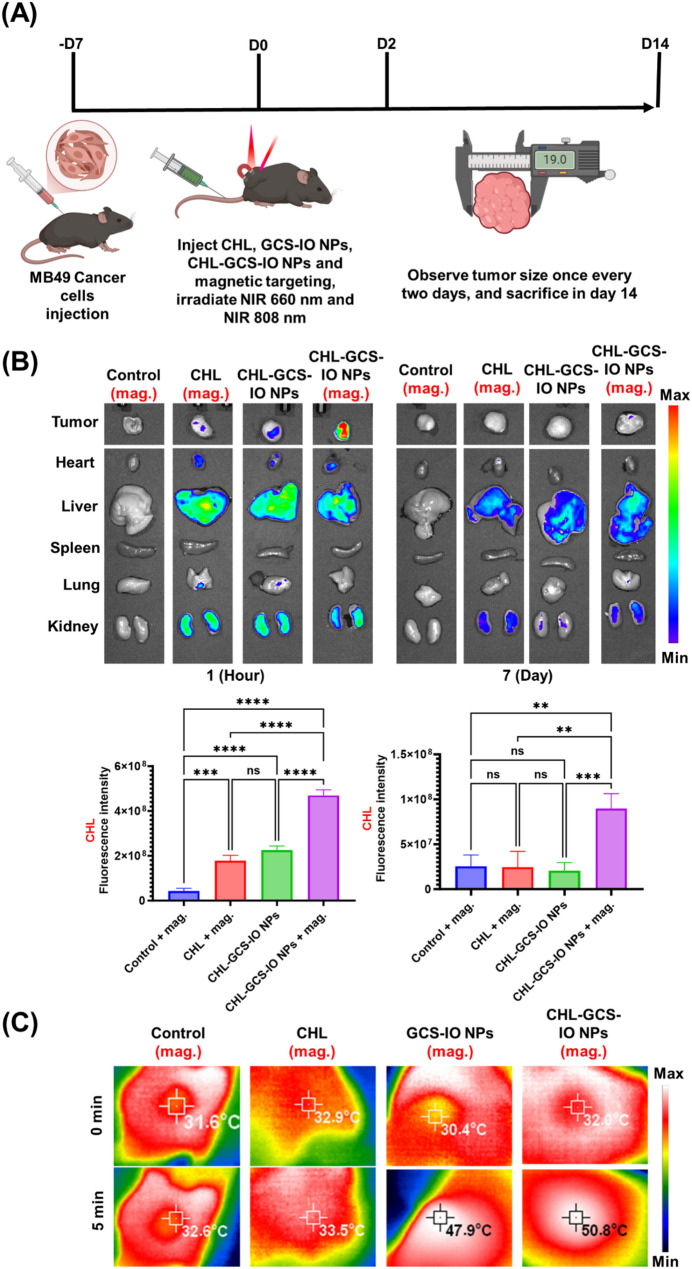
(**A**) Schematic timeline of animal experiments, including MB49 tumor cell injection, formulations administration, near infrared (NIR) irradiation, and tumor size monitoring. This image was created and edited using BioRender software. (**B**) Biodistribution of *Chlorella* (CHL) and CHL-glycol chitosan iron oxide (GCS-IO) NPs in MB49 tumor-bearing mice 1 h after an intravenous injection, both with and without magnetic targeting. Fluorescence intensity maps show the accumulation of CHL-GCS-IO NPs in tumors and organs (heart, liver, spleen, lungs, and kidneys). A quantitative analysis indicated a significant increase in tumor accumulation by CHL-GCS-IO NPs in the magnetic-targeted group compared to the other groups. Biodistribution of CHL and CHL-GCS-IO NPs 7 days post-injection and treatments. Fluorescence imaging demonstrated clearance of the formulations, with a residual signal observed in the tumor of CHL-GCS-IO NPs of the magnetic-targeted group. A quantitative analysis showed significantly higher retention in the tumor by magnetically targeted CHL-GCS-IO NPs. (**C**) Infrared thermal imaging of MB49 tumor-bearing mice subjected to 808-nm NIR irradiation for 5 min. Images reveal a significant temperature rise in the GCS-IO NPs and CHL-GCS-IO NPs groups, with CHL-GCS-IO NPs (magnetic) reaching the highest temperature (~ 50.8 °C), indicating a strong photothermal conversion efficacy

Encouraged by the promising in vitro results, we further expanded our investigation by conducting in vivo studies to rigorously evaluate the performance of our design in animal models, aiming to validate our key hypothesis. Figure 7A outlines the timeline for the animal experiments, detailing the sequence of MB49 tumor cell injection, formulations administration, NIR irradiation, and tumor size monitoring. The treatment period spanned from day 0 (injection of CHL, GCS-IO NPs, or CHL-GCS-IO NPs) to day 14 (sacrifice and final tumor size measurement). The biodistribution analysis presented in Fig. 7B evaluated the accumulation and clearance of CHL and CHL-GCS-IO NPs in major organs and tumors in MB49 tumor-bearing mice using in vivo imaging system (IVIS) imaging. In and 7B after an IV injection, CHL-GCS-IO NPs (with magnetic targeting) demonstrated the highest accumulation in the tumor site compared to the CHL and CHL-GCS-IO NPs groups without magnetic guidance. The fluorescence intensity in the tumor was significantly higher in the CHL-GCS-IO NPs group under magnetic guidance, indicating effective magnetic targeting and enhanced tumor retention of CHL-GCS-IO NPs. Fluorescence was also detected in the liver and kidney, consistent with typical polymer formulations biodistribution. A quantitative analysis of the fluorescence intensity confirmed a significant increase in tumor accumulation by the magnetically targeted CHL-GCS-IO NPs group, while other groups showed relatively lower tumor targeting. In contrast, the clearance of NPs after 7 days showed relatively minimal fluorescence in most organs and the tumor. The CHL-GCS-IO NPs group with magnetic targeting retained a higher residual fluorescence signal in the tumor compared to the CHL and other groups, although the overall signal had decreased, indicating effective metabolism and clearance of NPs from the system. At the 14th day of IVIS imaging (Fig. S1D), the different formulation-treated groups—Control (+ Magnet), CHL (+ Magnet), CHL-GCS-IO NPs, and CHL-GCS-IO NPs + Magnet—show no visible accumulation in major organs, including the heart, spleen, liver, lungs, and kidneys. This suggests that the formulations may have been effectively cleared from circulation. Figure 7C illustrates thermal imaging results of tumor-bearing mice subjected to 808-nm NIR irradiation. Thermal images reveal the photothermal effects of the different NP treatments. The control and CHL groups showed minimal temperature increases (~ 32 °C), indicating the absence of significant photothermal activity. In contrast, GCS-IO NPs and CHL-GCS-IO NPs (with magnetic targeting) exhibited substantial temperature increases, with CHL-GCS-IO NPs reaching over 50 °C after 5 min of NIR irradiation, confirming their superior photothermal conversion capability. The maximum temperature observed in the CHL-GCS-IO NPs group (50.8 °C) demonstrated their high efficiency for heat generation, which is critical for PTT applications. The ability to achieve such high temperatures within a short period is indicative of the strong photothermal response of the CHL-GCS-IO NPs, enhanced by the combination of magnetic targeting and NIR activation. This thermal rise is essential for inducing localized hyperthermia, which contributes to destruction of tumor cells in vivo. Together, these results confirmed the multifunctionality of CHL-GCS-IO NPs for magnetically guided targeting, efficient tumor accumulation, and potent photothermal effects under NIR irradiation. This system holds promise for future applications in magnetic-targeted PTT and other multimodal cancer therapies, particularly in overcoming challenges posed by tumor hypoxia and improving the precision of therapeutic interventions.

**Fig. 8:**
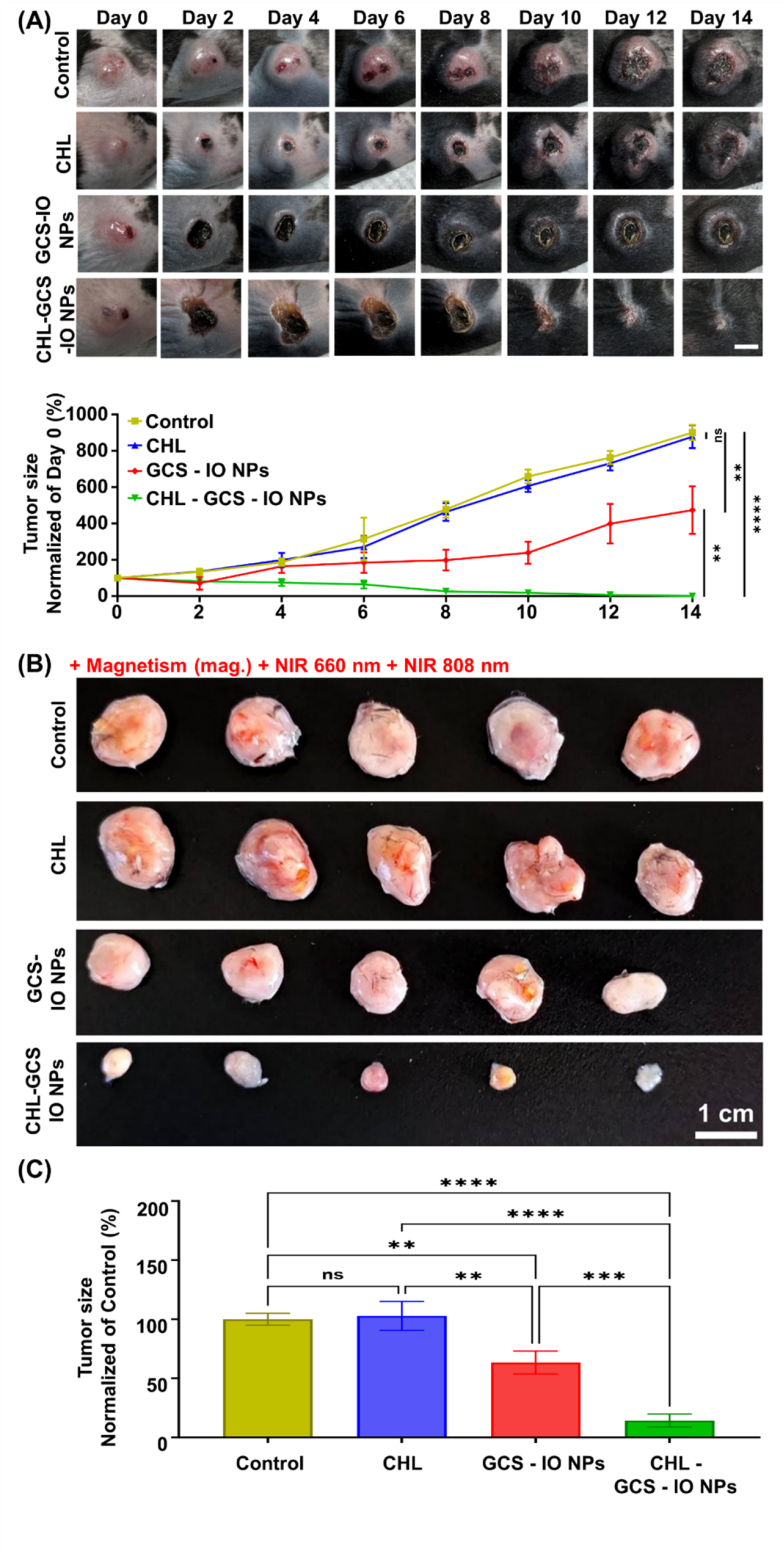
Antitumor efficacy of *Chlorella* (CHL), glycol chitosan iron oxide nanoparticles (GCS-IO NPs), and CHL-GCS-IO NPs in MB49 tumor-bearing mice with magnetic targeting and near infrared (NIR) irradiation. (**A**) Representative images of tumors in MB49 tumor-bearing mice treated with control, CHL, GCS-IO NPs, and CHL-GCS-IO NPs over a 14-day period. Tumor sizes were monitored and photographed every 2 days following intravenous administration of the formulations and subsequent magnetic targeting, and NIR 660- and 808-nm irradiation. Quantitative tumor growth curves for each group, normalized to the day 0 tumor size, are shown in the bottom panel. Data are presented as mean ± SD (*n* = 5). Scale bar: 0.5 cm. (**B**) Ex vivo images of excised tumors from all treatment groups after sacrifice on day 14. The tumor size and appearance visibly decreased in the GCS-IO NPs and CHL-GCS-IO NPs groups compared to the control and CHL groups, with the smallest tumor masses observed in the CHL-GCS-IO NPs group. (**C**) Quantitative analysis of sizes of excised tumors from all groups, normalized to control group's tumor size. Data are presented as the mean ± SD (*n* = 5)

The in vivo results in Fig. 8A illustrate the antitumor efficacy of CHL, GCS-IO NPs, and CHL-GCS-IO NPs in MB49 tumor-bearing mice following an *i.v.* injection and treatment with magnetic targeting and dual NIR irradiation (660 and 808 nm). Over the 14-day observation period, the control and CHL groups showed continued tumor growth, with visible expansion of the tumor site, as confirmed by photographic evidence. In contrast, the GCS-IO NPs and CHL-GCS-IO NPs groups exhibited significant tumor shrinkage, especially in the CHL-GCS-IO NPs group. Tumor growth in the CHL-GCS-IO NP group was effectively halted, with some tumors showing regression after treatment. The graphical representation of tumor size over time confirmed these observations, with the CHL-GCS-IO NPs group showing the most pronounced reduction in tumor size. Notably, magnetic targeting combined with the PTT and PST properties of CHL-GCS-IO NPs played a key role in enhancing antitumor efficacy compared to the other groups. Figure 8B and C further support the antitumor effects of the different treatments. At the time of sacrifice on day 14, control and CHL-treated tumors appeared significantly larger than those treated with GCS-IO NPs and CHL-GCS-IO NPs. Images of excised tumors clearly show that the CHL-GCS-IO NPs group had the smallest residual tumors, indicating strong therapeutic effects when magnetic targeting and NIR irradiation were applied. Quantitative analysis of excised tumor weights (Fig. 8C) reinforced these findings, with the CHL-GCS-IO NPs group showing a statistically significant reduction in tumor size compared to all other groups. The GCS-IO NPs group also demonstrated antitumor effects, but these were less pronounced than those of the CHL-GCS-IO NPs group, highlighting the critical role of photosynthetic oxygenation and synergistic effects of CHL in enhancing the efficacy of the NP system.

The enhanced antitumor activity observed in the CHL-GCS-IO NPs group can be attributed to the combination of multiple therapeutic modalities—magnetically guided targeting, PTT, ferroptosis, and PST. The presence of CHL as a photosynthetic agent likely contributed to the increased oxygen levels within the TME, helping to alleviate hypoxia and improve the efficacy of PST. The significant photothermal effect generated by NIR irradiation, as demonstrated by the temperature rise in previous figures, further amplified the therapeutic response, contributing to effective tumor ablation. In summary, Fig. 8 demonstrates the superior antitumor efficacy of the CHL-GCS-IO NPs system under combined magnetic targeting, NIR irradiation, and multimodal therapy. The ability of this system to halt tumor growth and induce significant tumor regression highlights its potential as a powerful therapeutic tool for cancer treatment. Results suggest that the combination of magnetotactic-like, photothermal, and photosynthetic effects offers a promising strategy for addressing challenges posed by the TME, including hypoxia, and improving therapeutic outcomes in cancer treatment.

Results from Figure S2 provide a detailed histological analysis of tumor and soft-tissue organs following administration of CHL, GCS-IO NPs, and CHL-GCS-IO NPs, with magnetic targeting and dual NIR irradiation (660 and 808 nm). Hematoxylin and eosin (H&E) staining was utilized to evaluate tissue morphology and potential damage caused by the treatments. The outcomes offer insights into the therapeutic effects and biocompatibility of the CHL-GCS-IO NPs system. H&E staining (Figure S2A) of tumor tissues revealed distinct morphological differences between the treatment groups. In the control group, tumor tissues maintained a highly proliferative architecture, with densely packed cells and minimal necrosis. In contrast, the CHL-treated group showed moderate signs of necrosis but retained a considerable amount of viable tumor cells, indicating a limited therapeutic effect. The GCS-IO NPs group exhibited more-extensive necrosis and a reduction in overall tumor cell density, likely due to the combined effects of magnetic targeting, PTT, and ferroptosis.

The most pronounced effects were observed in the CHL-GCS-IO NPs group, where widespread necrosis and a significant reduction in tumor cell density were evident. Tumor tissues in this group displayed a highly disrupted architecture with minimal viable cells, suggesting that the combined effects of PST, PTT, ferroptosis, and magnetic targeting effectively induced tumor cell death. Photosynthetic oxygenation provided by CHL likely contributed to alleviating hypoxia within the TME, thereby enhancing the efficacy of PST. These results highlight the superior antitumor efficacy of the CHL-GCS-IO NPs system under multimodal treatment conditions. H&E staining of major organs (Figure S2B), including the heart, liver, spleen, lungs, and kidneys, was performed to assess the potential off-target toxicity of formulationss treatments. The negative control group, representing healthy tissues, displayed a normal histological architecture in all organs. In the control and others-treated groups, organ tissues showed minimal changes, indicating that the tumor growth and CHL administration alone did not induce significant systemic toxicity. Overall, results from Figure S2 indicate that the CHL-GCS-IO NPs system effectively induced tumor necrosis without causing significant damage to healthy tissues. This biocompatibility, combined with the enhanced therapeutic efficacy, makes CHL-GCS-IO NPs a promising candidate for multimodal cancer therapy.

**Fig. 9:**
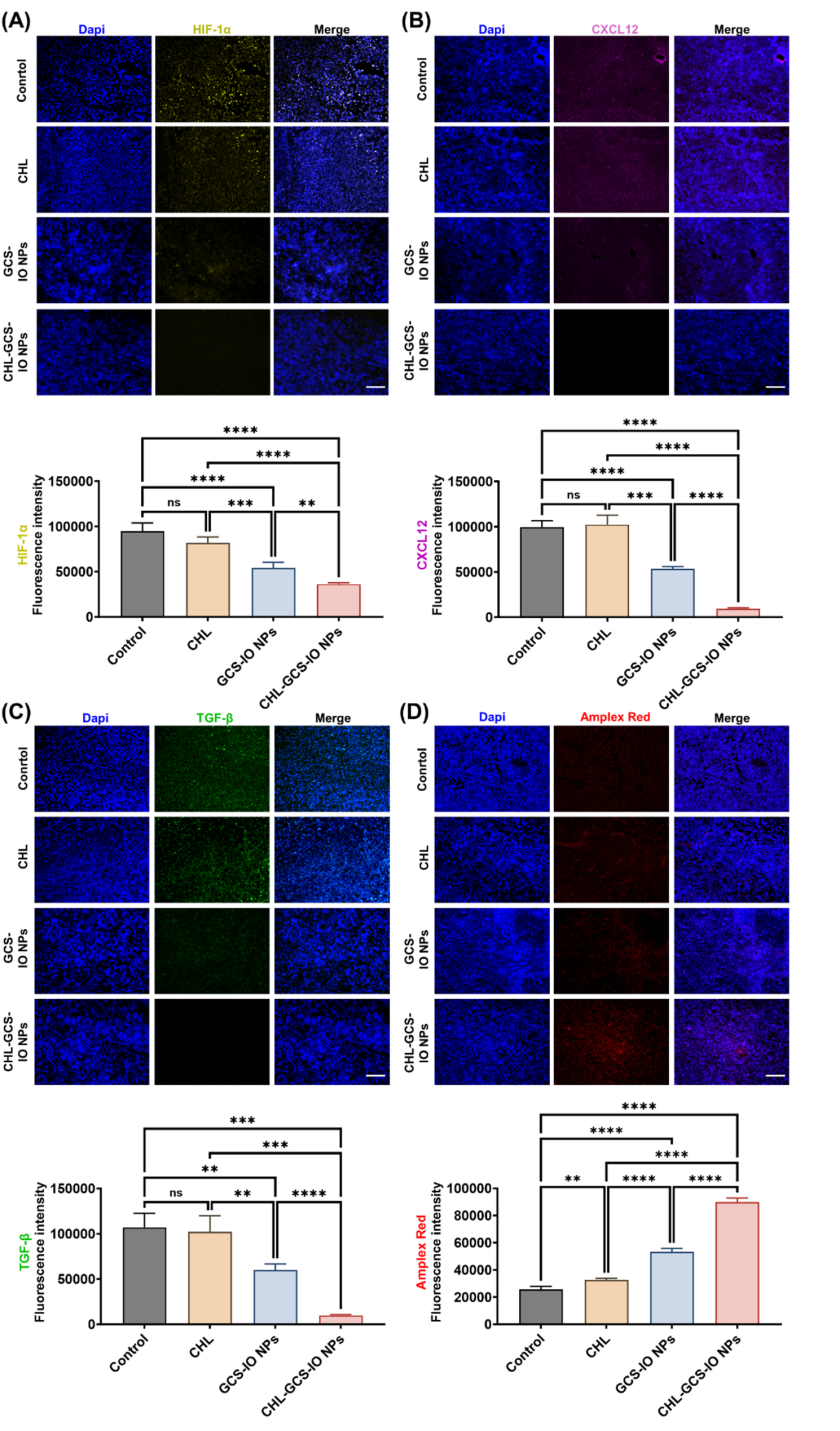
Immunofluorescence (IF) analysis of key tumor markers in MB49 tumor tissues after treatment with *Chlorella* (CHL), glycol chitosan iron oxide nanoparticles (GCS-IO NPs), and CHL-GCS-IO NPs under magnetism and dual near infrared (NIR) irradiation (660 + 808 nm). (**A**) Hypoxia-inducible factor (HIF)-1α staining (yellow). IF images of tumor sections stained for HIF-1α, a marker of hypoxia, showing higher expression in the control and CHL-treated groups. A significant reduction in HIF-1α levels was observed in the GCS-IO NPs group, while the CHL-GCS-IO NPs group exhibited nearly undetectable levels. Quantification of the fluorescence intensity revealed significant downregulation of HIF-1α in the CHL-GCS-IO NPs group. (**B**) C-X-C Motif Chemokine Ligand 12 (CXCL12) (purple). IF images showed CXCL12 expression, a factor linked to tumor metastasis and angiogenesis. The control group showed high CXCL12 expression, which was moderately reduced in the CHL and GCS-IO NPs groups. The CHL-GCS-IO NPs group showed the lowest expression, suggesting suppression of proangiogenic signaling. Quantification indicated significant downregulation in the CHL-GCS-IO NPs group. (**C**) Transforming growth factor (TGF)-β staining (green). IF images of tumor sections stained for TGF-β, a cytokine involved in immune suppression and tumor metastasis. The control group showed high TGF-β expression, which slightly decreased in the CHL and GCS-IO NPs groups. The CHL-GCS-IO NPs group showed the most substantial reduction in TGF-β levels. Quantification confirmed significant downregulation of TGF-β in the CHL-GCS-IO NPs group. (**D**) Amplex red staining (red). Detection of reactive oxygen species (ROS) via Amplex red staining. The control group showed low ROS levels, while the CHL and GCS-IO NPs groups demonstrated moderate ROS production. The CHL-GCS-IO NPs group showed the highest ROS levels, indicating magnetic navigation with strong photosynthetic oxygen generation under NIR irradiation. A quantitative analysis confirmed significantly higher ROS production in the CHL-GCS-IO NPs group. Scale bar: 100 μm. Statistical significance: ns, not significant, ** *p* < 0.01, *** *p* < 0.001, **** *p* < 0.0001

Fluorescence microscopic data (Fig. 9A) showed immunofluorescence (IF) staining of hypoxia-inducible factor (HIF)-1α, a key marker of hypoxia [58] and which plays a critical role in tumor progression and therapy resistance. The control group demonstrated strong HIF-1α expression, consistent with the hypoxic conditions typically observed within the TME. The CHL-treated group showed partial HIF-1α reduction, though it was moderately reduced compared to the control. In contrast, the GCS-IO NPs group also exhibited a decrease in HIF-1α expression, suggesting partial alleviation of tumor hypoxia due to PTT. Notably, the CHL-GCS-IO NPs group showed the lowest HIF-1α expression, with nearly undetectable levels of fluorescence intensity. This indicated that photosynthetic oxygenation provided by magnetotactic-like CHL-GCS-IO NPs effectively reduced hypoxia in the tumor, further supporting the enhanced therapeutic response seen in this group. The quantitative analysis (by ImageJ software) confirmed the significant downregulation of HIF-1α in the CHL-GCS-IO NPs group, which was significantly lower than in the other treatment groups.

We focused on CXCL12 (Fig. 9B), a chemokine closely associated with tumor metastasis activity and angiogenesis [59]. Similar to HIF-1α, the control group showed high CXCL12 expression, while the CHL-treated group exhibited slightly reduced levels. The GCS-IO NPs group demonstrated a further decrease in CXCL12 expression, likely due to the effects of PTT and ferroptosis. The most substantial reduction was observed in the CHL-GCS-IO NPs group, where CXCL12 expression was significantly downregulated. This suggested that the multimodal therapeutic approach not only targeted the primary tumor but may also have suppressed metastatic processes by reducing proangiogenic signals. A quantitative analysis confirmed that the CHL-GCS-IO NPs group exhibited the lowest CXCL12 levels, significantly lower than those in the other groups, highlighting the role of CHL-GCS-IO NPs in inhibiting factors that promote tumor progression and metastasis. We further evaluated transforming growth factor (TGF)-β (Fig. 9C), a cytokine involved in immune suppression and tumor metastasis [60]. The control group displayed high TGF-β expression, indicative of an immunosuppressive TME that favors tumor growth. The CHL group showed moderate TGF-β expression, while the GCS-IO NPs group exhibited a significant reduction in TGF-β levels. The CHL-GCS-IO NPs group, however, demonstrated the most profound reduction in TGF-β expression, suggesting that the combined effects of magnetic navigation with photosynthetic oxygenation and PTT induced tumor cell death and also reprogrammed the TME to a more immunologically favorable state. This shift may enhance immune-mediated tumor eradication by mitigating immune suppression. A quantitative analysis revealed that the CHL-GCS-IO NPs group had significantly lower TGF-β expression compared to all other groups. Fluorescence data (Fig. 9D) were used to detect ROS via Amplex red staining [61]; the generation of ROS is related to photosynthetic oxygen produced by CHL under light irradiation. The control group showed low ROS levels, while the CHL-treated group demonstrated moderate ROS production due to photosynthetic activity. The GCS-IO NPs group showed a further increase in ROS levels, likely due to the combined effects of ferroptosis, PTT- and PST-assisted cancer cellular ROS generation. The CHL-GCS-IO NPs group, however, exhibited the highest ROS production (*p* < 0.0001), confirming the strong magnetic navigation with photosynthetic activity of CHL in this group, which was further amplified by magnetic targeting and NIR irradiation. ROS generation contributed to enhanced tumor cell death and the overall therapeutic efficacy of the CHL-GCS-IO NPs system. A quantitative analysis corroborated these findings, with the CHL-GCS-IO NPs group showing significantly higher ROS levels than all other groups.

**Fig. 10:**
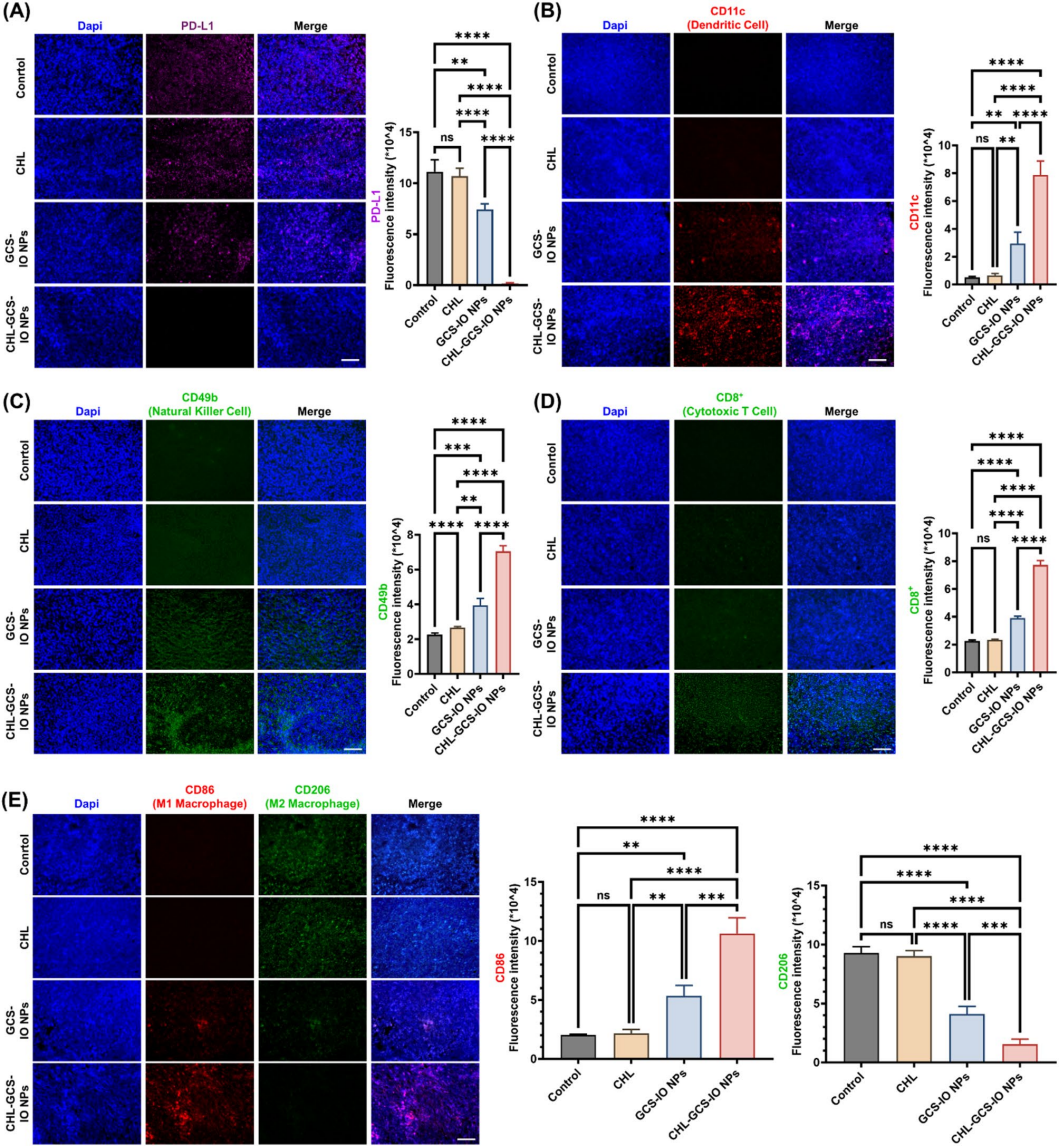
Immunomodulatory effects of *Chlorella* (CHL), glycol chitosan iron oxide nanoparticles (GCS-IO NPs), and CHL-GCS-IO NPs in tumor-bearing mice. Immunofluorescence (IF) staining and quantitative analysis of immune markers in MB49 tumor tissues after treatment with CHL, GCS-IO NPs, and CHL-GCS-IO NPs under magnetic targeting and dual near infrared (NIR) irradiation (660 and 808 nm). (**A**) Programmed death ligand 1 (PD-L1) expression. PD-L1 IF staining (purple) in tumor tissues showed reduced expression in the GCS-IO NPs and CHL-GCS-IO NPs groups, with the most significant reduction in the CHL-GCS-IO NPs group. A quantitative analysis confirmed significantly lower PD-L1 levels in the CHL-GCS-IO NPs group compared to all other groups. (**B**) Cluster of differentiation 11c (CD11c) expression (dendritic cells (DCs)). CD11c IF staining (red) in tumor tissues indicated enhanced DC activation in the GCS-IO NPs group, with the highest activation seen in the CHL-GCS-IO NPs group. (**C**) CD49b expression (natural killer (NK) cells). CD49b IF staining (green) revealed increased NK cell recruitment and activation, particularly in the CHL-GCS-IO NPs group, where the highest expression of CD49b was observed. (**D**) CD8^+^ T-cell infiltration. CD8^+^ IF staining (green) showed robust T-cell infiltration in the CHL-GCS-IO NPs group, with significantly higher CD8^+^ levels compared to all other groups. (**E**) Macrophage polarization. CD86 (M1 macrophages, red) and CD206 (M2 macrophages, green) staining showed a marked shift toward proinflammatory M1 polarization in the CHL-GCS-IO NPs group, with a significant increase in CD86 and a corresponding decrease in CD206 expression. Scale bars: 100 μm. A quantitative analysis was performed using ImageJ software, and data are presented as the mean ± SD. Statistical significance: ns, not significant; ** *p* < 0.01; *** *p* < 0.001; **** *p* < 0.0001

Data from Fig. 10 provide an insightful exploration of immune modulation induced by the CHL-GCS-IO NPs system, as demonstrated through IF staining of various key immune markers within the TME. Programmed death-ligand 1 (PD-L1; Fig. 10A), a major checkpoint protein often associated with immune evasion in cancer [62], was markedly overexpressed in the control group, indicating a highly immunosuppressive TME. PD-L1 expression was reduced in the CHL-treated group, showing some immune modulation, although the change was not substantial. In contrast, the GCS-IO NPs group exhibited a significant reduction in PD-L1 expression, likely due to the effects of magnetic navigation with PTT and ferroptosis, which disrupted tumor immune evasion. However, the most dramatic decrease in PD-L1 was observed in the CHL-GCS-IO NPs group (*p* < 0.0001), where the lowest fluorescence intensity was recorded. This indicated that magnetic navigation with multimodal treatment (PST, PTT, and ferroptosis) not only enhanced the antitumor efficacy but also suppressed immune checkpoint mechanisms, potentially allowing for greater immune system engagement against the tumor. Ferroptosis, an iron-dependent cell death driven by lipid peroxidation, is influenced by hypoxia Notably, hypoxia can influence PD-L1 expression, which has been linked to ferroptosis regulation. While hypoxia may promote ferroptosis through lipid peroxidation, it can also inhibit it via PD-L1 and GPX4 upregulation. This interplay is crucial in ischemic diseases, cancer, and immune regulation, making ferroptosis a key therapeutic target in hypoxia-related conditions [63, 64]. A quantitative analysis confirmed that PD-L1 levels in the CHL-GCS-IO NPs group were significantly lower than in all other groups, underlining the potent immunomodulatory effects of this treatment.

CD11c (Fig. 10B) is a marker of dendritic cells (DCs), which play pivotal roles in antigen presentation and activation of T cells. In the control group, CD11c expression was minimal, reflecting a lack of immune activation. The CHL-treated group showed a slight increase in CD11c expression, but it remained relatively low. In contrast, the GCS-IO NPs group demonstrated marked upregulation of CD11c, indicating that the treatment had begun to activate immune responses, likely driven by the magnetic navigation with inflammatory and immunogenic effects of PTT and ferroptosis. However, the highest CD11c expression was found in the CHL-GCS-IO NPs group, suggesting that the combined therapeutic magnetic navigation with modalities not only induced potent antitumor effects but also significantly enhanced DC activation. This increase in CD11c-positive cells was crucial for promoting tumor antigen presentation and initiating a strong adaptive immune response. The quantitative analysis (by ImageJ software) further supported this, showing a significant upregulation of CD11c in the CHL-GCS-IO NPs group compared to all other treatments.

Natural killer (NK) cells, marked by CD49b expression (Fig. 10C), are key players in the innate immune system and are responsible for directly killing tumor cells [66]. In the control group, CD49b expression was minimal, indicating a lack of NK cell activity. The CHL-treated group showed a slight increase in NK cell activation, while the GCS-IO NPs group exhibited a more-substantial rise in CD49b expression, reflecting enhanced innate immune activity, likely stimulated by magnetic navigation with PTT and ferroptosis. The most pronounced NK cell activation was observed in the CHL-GCS-IO NPs group, where the fluorescence intensity was significantly higher than in the other groups. This suggested that the magnetic navigation with multimodal therapy effectively recruited and activated NK cells, which may play a critical role in the rapid destruction of tumor cells. A quantitative analysis revealed a significant increase in CD49b-positive NK cells in the CHL-GCS-IO NPs group compared to all other treatments, highlighting the potent immune-stimulating effects of this system.

CD8^+^ T cells (Fig. 10D) are crucial in the adaptive immune response, particularly in targeting and killing tumor cells [67]. In the control group, CD8^+^ expression was low, indicating poor T-cell infiltration into the tumor. The CHL-treated group showed moderate CD8^+^ T cell activation, while the GCS-IO NPs group displayed a substantial increase in the CD8^+^ T-cell presence, suggesting that the treatment promoted cytotoxic T-cell recruitment and activation. The CHL-GCS-IO NPs group showed the highest expression of CD8^+^ T cells, indicating robust T-cell-mediated antitumor immunity. This suggested that the multimodal therapy not only induced direct tumor cell death but also enhanced the recruitment and activation of cytotoxic T cells, which can further contribute to long-term tumor control. A quantitative analysis confirmed that CD8^+^ T cell levels were significantly higher in the CHL-GCS-IO NPs group compared to all other treatments, demonstrating the powerful immune-stimulating potential of this system.

Macrophages (Fig. 10E) can polarize into either the proinflammatory M1 phenotype, which promotes antitumor immunity, or the immunosuppressive M2 phenotype, which supports tumor progression [68]. The control group showed high CD206 (M2 macrophage) expression, consistent with an immunosuppressive TME. The CHL-treated group showed a slight decrease in CD206 expression, while the GCS-IO NPs group exhibited a marked reduction in M2 macrophages, with a corresponding increase in CD86 (M1 macrophage) expression. However, the most profound shift in macrophage polarization was seen in the CHL-GCS-IO NPs group, where M1 macrophages were significantly upregulated, and M2 macrophages were substantially downregulated. This indicated that the multimodal therapy not only disrupted the tumor-supportive functions of M2 macrophages but also promoted activation of M1 macrophages, which are essential for initiating and sustaining antitumor immune responses. A quantitative analysis (by ImageJ software) further supported this, showing a significant increase in CD86 (M1) and a corresponding decrease in CD206 (M2) in the CHL-GCS-IO NPs group compared to all other treatments. In summary, the data presented in Fig. 10 underscore the comprehensive immune-modulating effects of the CHL-GCS-IO NPs system. By downregulating immunosuppressive mechanisms (e.g., PD-L1 and M2 macrophages) and upregulating immune-stimulating factors (e.g., CD11c, CD49b, CD8^+^, and M1 macrophages), this system fostered a potent antitumor immune response. The combination of PST, PTT, and ferroptosis not only induced direct tumor ablation but also reprogrammed the TME to favor immune activation, offering a promising strategy for effective cancer immunotherapy.

## Discussion

The present study demonstrates the potent theranostic potential of CHL-GCS-IO NPs, magnetotactic-like system, for MB49 cancer treatment, leveraging multimodal therapeutic strategies including magnetism, PTT, PST, and immune modulation. The findings underscore the superior antitumor efficacy of CHL-GCS-IO NPs, which integrate magnetic targeting, photosynthetic oxygen production, and the ability to modulate the TME to alleviate hypoxia and enhance immune responses.

The CHL-GCS-IO NPs exhibited excellent photothermal properties under NIR irradiation, efficiently raising the tumor temperature above 50 °C, which is sufficient to induce hyperthermia and tumor cell ablation. The magnetic responsiveness of the IO NPs allowed precise tumor targeting, minimizing off-target effects and enhancing accumulation at the tumor site. The superparamagnetic nature of the GCS-IO NPs ensured minimal residual magnetization, reducing the risk of systemic side effects associated with magnetic NPs. The combination of PTT and PST addressed the limitations of conventional therapies, such as poor tumor oxygenation and resistance to treatments, by continuously generating oxygen in the hypoxic TME. This alleviation of hypoxia improved the efficacy of both PTT and PST, contributing to enhanced ROS generation and tumor cell death.

One of the key findings is the strong immune modulatory effect of magnetotactic-like CHL-GCS-IO NPs, which significantly downregulated PD-L1 expression and enhanced activation of immune cells, such as DCs, NK cells, and CD8^+^ T cells. The significant reduction in PD-L1 expression suggested that CHL-GCS-IO NPs can suppress immune evasion mechanisms commonly employed by tumors, facilitating the recruitment and activation of cytotoxic immune cells. The increased infiltration of CD8^+^ T cells, coupled with the reprogramming of macrophages from an M2 (tumor-promoting) to M1 (tumor-suppressing) phenotype, further emphasizes the potential of CHL-GCS-IO NPs to reshape the immunosuppressive TME and stimulate a robust antitumor immune response.

Compared to single-modality treatments, CHL-GCS-IO NPs provide a comprehensive therapeutic approach by addressing multiple challenges associated with tumor growth and resistance. Alleviating hypoxia through PST directly combats one of the primary causes of treatment resistance, while PTT induces direct tumor ablation. The ability of CHL-GCS-IO NPs to modulate the immune system, particularly through reducing PD-L1 expression and activating CD8^+^ T cells, highlights their potential to overcome immune resistance and promote long-term antitumor immunity. This multimodal strategy not only enhanced immediate tumor clearance but may also contribute to immune memory, preventing future tumor recurrence.

The unique design of artificial intelligence (AI)-conceptualized CHL-GCS-IO NPs leverages a multimodal approach to tackle complexities of the TME, particularly addressing hypoxia, immune evasion, and resistance to traditional therapies. By integrating ferroptosis, PST, and PTT, this system not only induced direct tumor cell death but also reprogrammed the TME to support robust immune activation. Ferroptosis, an iron-dependent form of programmed cell death characterized by lipid peroxidation [38], played a critical role in the anticancer effects of CHL-GCS-IO NPs. IO NPs facilitate this process by catalyzing the Fenton reaction, leading to the generation of ROS and oxidative stress, particularly under hypoxic conditions typical of the TME. The induction of ferroptosis disrupts the tumor’s lipid metabolism, causing tumor cell death, which in turn promotes the release of tumor-associated antigens that can be captured by DCs. Upregulation of CD11c expression observed in this study suggested that the ferroptosis-mediated release of damage-associated molecular patterns (DAMPs) enhanced DC activation [69], a key step in initiating adaptive immune responses. Activated DCs can efficiently present tumor antigens to CD8^+^ T cells, promoting a cytotoxic T-cell response that targets and destroys tumor cells.

PTT, driven by heat generated through NIR laser irradiation, induces localized hyperthermia, leading to tumor cell apoptosis and necrosis. The thermal damage inflicted by PTT also results in the release of tumor antigens, which further stimulates the recruitment of immune cells to the TME. In particular, PTT enhanced the infiltration of CD8^+^ cytotoxic T cells [70], as observed by the significant upregulation of CD8^+^ markers in the CHL-GCS-IO NPs-treated groups. These CD8^+^ T cells play a crucial role in directly attacking tumor cells, particularly when immune checkpoint inhibitors like PD-L1 are suppressed. In this study, the marked reduction in PD-L1 expression in CHL-GCS-IO NPs-treated tumors suggested that PTT, in combination with ferroptosis, disrupted immune tolerance, allowing for more-effective CD8^+^ T-cell-mediated tumor cell killing.

PTT also affects NK cells, known for their ability to directly lyse tumor cells without the need for prior antigen exposure [71]. Upregulation of CD49b (a marker of NK cells) in the CHL-GCS-IO NPs group indicated that the thermal and oxidative stress induced by PTT may have enhanced NK cell recruitment and activation. NK cells, like CD8^+^ T cells, play a critical role in controlling tumor growth, especially in immunologically “cold” tumors, which are typically resistant to conventional immunotherapies.

A major challenge in cancer therapy is the presence of hypoxia within the TME, which promotes treatment resistance and immune suppression. CHL-GCS-IO NPs, through the incorporation of the photosynthetic *Chlorella* (CHL), generated oxygen under light irradiation, alleviating tumor hypoxia. The reduction in hypoxia, as evidenced by the decreased expression of HIF-1α in the CHL-GCS-IO NPs group, directly correlated with improved tumor oxygenation and increased therapeutic efficacy. Alleviation of hypoxia not only synergized ferroptosis and PTT but also reprogrammed the TME to be more favorable for immune cell infiltration and activation.

Hypoxia is also known to promote the polarization of tumor-associated macrophages (TAMs) toward an M2 immunosuppressive phenotype, which supports tumor growth and immune evasion [72]. By alleviating hypoxia, CHL-GCS-IO NPs shifted TAM polarization toward the M1 proinflammatory phenotype, as indicated by upregulation of CD86 (an M1 marker) and downregulation of CD206 (an M2 marker) [73]. This M1 shift is critical for enhancing antitumor immunity, as M1 macrophages secrete proinflammatory cytokines that recruit and activate CD8^+^ T cells and NK cells [74].

The observed downregulation of PD-L1 expression in the CHL-GCS-IO NPs group was particularly significant. PD-L1 is a key immune checkpoint protein that tumors use to evade immune detection by inhibiting the activity of CD8^+^ T cells [74]. Suppression of PD-L1, likely facilitated by the combined effects of ferroptosis, PTT, and anti-hypoxia therapy [75–77], reactivated CD8^+^ T cells, allowing more-effective immune-mediated tumor cell destruction. This effect, combined with the enhanced activation of DCs and NK cells, suggested that drug-free CHL-GCS-IO NPs not only served as direct tumor killers but also functioned as powerful immune modulators.

The integration of ferroptosis, PTT, and PST in CHL-GCS-IO NPs addresses critical aspects of tumor biology, particularly hypoxia, immune evasion, and resistance to treatment. By inducing direct tumor cell death, modulating the immune microenvironment, and alleviating hypoxia, this NP system demonstrated a comprehensive therapeutic strategy. The combination of ferroptosis with immune checkpoint inhibition (PD-L1 downregulation) and activation of key immune cells, such as DCs, NK cells, and CD8^+^ T cells, provides a strong basis for further exploration of this system in cancer immunotherapy.

Previous studies [Photosynthetic Tumor Oxygenation by Photosensitized Cyanobacterial Cells for Enhanced Photodynamic Therapy] have explored the use of photosynthetic microorganisms like cyanobacteria to enhance tumor oxygenation. This approach has been integrated into Type-II photodynamic therapy (PDT) to improve oxygen supply and, in turn, enhance reactive oxygen species (ROS) production.

However, our current study builds upon these existing methods by introducing a multimodal approach using magnetically guided Chlorella-based system (CHL-GCS-IO NPs). This advanced system not only leverages photosynthetic oxygenation but also incorporates magnetic targeting, photothermal therapy (PTT), and ferroptosis to address the persistent challenges of hypoxia, immune evasion, and drug resistance in solid tumors. The Chlorella microalgae in our system not only supply a sustained oxygen source but also contribute to immune activation by promoting macrophage polarization and alleviating the immunosuppressive effects in the tumor TME. The combined approach of ferroptosis, immune checkpoint inhibition (via PD-L1 downregulation), and activation of critical immune cells such as dendritic cells (DCs), natural killer (NK) cells, and CD8 + T cells forms a compelling foundation for future exploration in cancer immunotherapy.

This dual role of Chlorella—acting both as a therapeutic agent and an immune system activator—represents a novel concept that has not been comprehensively explored in prior research.

Moreover, by incorporating glycol chitosan (GCS) and iron oxide nanoparticles (IO NPs) into the Chlorella structure, our system benefits from enhanced biocompatibility and targeted delivery, reducing clearance rates and improving therapeutic efficiency. This nano-orchestrated algalrobot platform represents a highly sophisticated and integrated solution to overcome the limitations of previous strategies, which often relied on passive oxygenation or single-modality treatments.

Our study signifies a significant advancement over previous efforts by combining photosynthetic oxygenation, magnetic targeting, and ferroptosis, thus offering a more effective and sustainable method for addressing the hypoxic tumor microenvironment—a well-known challenge in cancer treatment.

CHL-GCS-IO NPs provide a comprehensive approach that addresses major challenges in bladder cancer treatment, such as hypoxia, immune evasion, and drug resistance. The system effectively reprogrammed the TME by alleviating hypoxia, enhancing immune cell infiltration, and shifting macrophage polarization from the immunosuppressive M2 phenotype to the proinflammatory M1 phenotype. This modulation is key for both tumor clearance and long-term immune surveillance. The significant downregulation of PD-L1, coupled with the enhanced activation of immune cells, like DCs, CD8^+^ T cells, and NK cells, positions CHL-GCS-IO NPs as a potent immune modulator that can potentially be combined with other immunotherapies. The photothermal ablation capabilities combined with IO-driven ferroptosis create a potent mechanism of tumor destruction. The ability to induce both thermal damage and oxidative stress in tumor cells enhances the system’s overall efficacy. The use of the photosynthetic *Chlorella* for continuous oxygen production in hypoxic tumor environments is a novel strategy to improve the effectiveness of therapies like PTT and ferroptosis. This photosynthesis-driven oxygenation alleviated one of the most significant barriers to effective cancer treatment. The robust immune activation achieved through the multimodal approach suggests the potential for the system to establish long-term immune memory, reducing the risk of tumor recurrence. The study highlighted minimal toxicity to healthy tissues, suggesting that CHL-GCS-IO NPs can offer a safe and biocompatible platform for future clinical applications. The multifunctionality of the system—combining therapeutic actions (PTT, PST, and ferroptosis) with diagnostic capabilities (MRI contrast)—provides a strong case for the system’s utility in both treating and monitoring tumor progression in real-time.

## Conclusions

The CHL-GCS-IO NPs system developed in this study presents a promising multimodal platform for cancer therapy by integrating photosynthetic oxygenation (PST), PTT, and ferroptosis. This formulation exhibited strong tumor-targeting abilities, magnetic responsiveness, and significant immunomodulatory effects within the TME. The combination of photothermal ablation, immune activation, and alleviation of hypoxia resulted in notable antitumor efficacy in MB49 tumor-bearing mice, as suggested by downregulation of immunosuppressive markers (e.g., PD-L1 and M2 macrophages) and upregulation of immune-stimulatory markers (e.g., CD8^+^ T cells, DCs, NK cells, and M1 macrophages).

This development offers a comprehensive strategy for combating tumor growth and overcoming therapy resistance, particularly in hypoxic environments. The system effectively reprogrammed the TME to favor immune activation, enhancing the recruitment and activity of DCs, cytotoxic T cells, NK cells, and M1 macrophages. This immune-stimulating effect is crucial for long-term tumor control and potential resistance against recurrence. The incorporation of IO NPs provided magnetic responsiveness, improving tumor accumulation and allowing precise control over NP distribution through an external magnetic field. The CHL-GCS-IO NPs system showed minimal toxicity to healthy tissues, demonstrating excellent biocompatibility while preserving the therapeutic effects. While magnetic targeting improved tumor accumulation, the biodistribution of NPs in non-targeted organs (e.g., liver and spleen) still presents a challenge, highlighting the need for further optimization of clearance mechanisms to reduce potential off-target effects.

Future research should focus on enhancing the system’s clinical applicability by developing more-efficient delivery methods, improving NIR light penetration, and optimizing NP dosing strategies to ensure maximal therapeutic impacts with minimal side effects. The CHL-GCS-IO NPs system’s multimodal approach can be extended to treat other types of solid tumors, especially those characterized by hypoxia and immune evasion. Further preclinical and clinical studies are necessary to validate its effectiveness across a broader spectrum of cancers. Combining this NP platform with other therapeutic modalities, such as chemotherapy, immunotherapy, or radiotherapy, could offer synergistic effects, enhancing overall treatment efficacies and reducing the likelihood of tumor recurrence. Future iterations of this system could incorporate smart, stimulus-responsive mechanisms (e.g., pH-sensitive or temperature-sensitive elements) to enhance drug release control, further improving the precision and efficacy of cancer treatment. In conclusion, the CHL-GCS-IO NPs system offers a multifaceted approach to tackle challenges posed by the TME, providing a robust platform for future cancer therapies. However, addressing current limitations and further exploring synergistic combinations with existing treatments will be essential for optimizing its clinical potential.

## Electronic supplementary material

Below is the link to the electronic supplementary material.

Supplementary Material 1

## Data Availability

No datasets were generated or analysed during the current study.
