# Supplementary material for: Nano-orchestrated magnetotactic-like navigation for electromagnetic theranostics and immune enhancement via photoautotrophic oxygenation, mild hyperthermia, and ferroptosis
Source: J Nanobiotechnology. 2025 Jun 13;23:442. doi: 10.1186/s12951-025-03488-7 (PMC12164064; doi:10.1186/s12951-025-03488-7)
Supplement: Supplementary file 1 — Supplementary Material 1 [file 12951_2025_3488_MOESM1_ESM.docx]

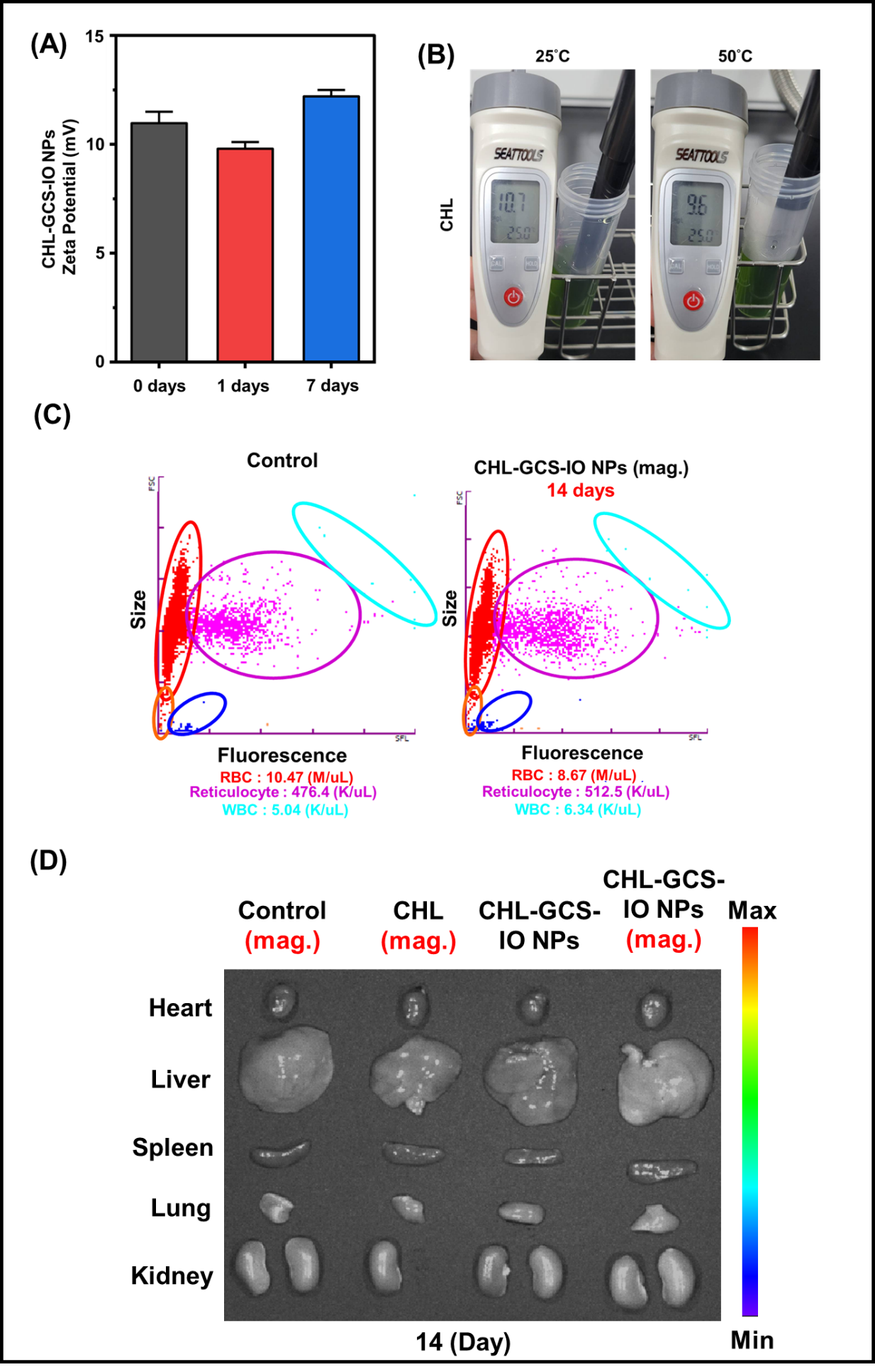


**Figure S1 :** (A) CHL-GCS-IO NPs Zeta potential for 0 days, 1 days, 7 days. (B) the photosynthetic oxygen production of CHL at different temperature. (C) Heamocompatibility data of control, CHL-GCS-IO NPs with Mag. (D) IVIS data at 14 days.


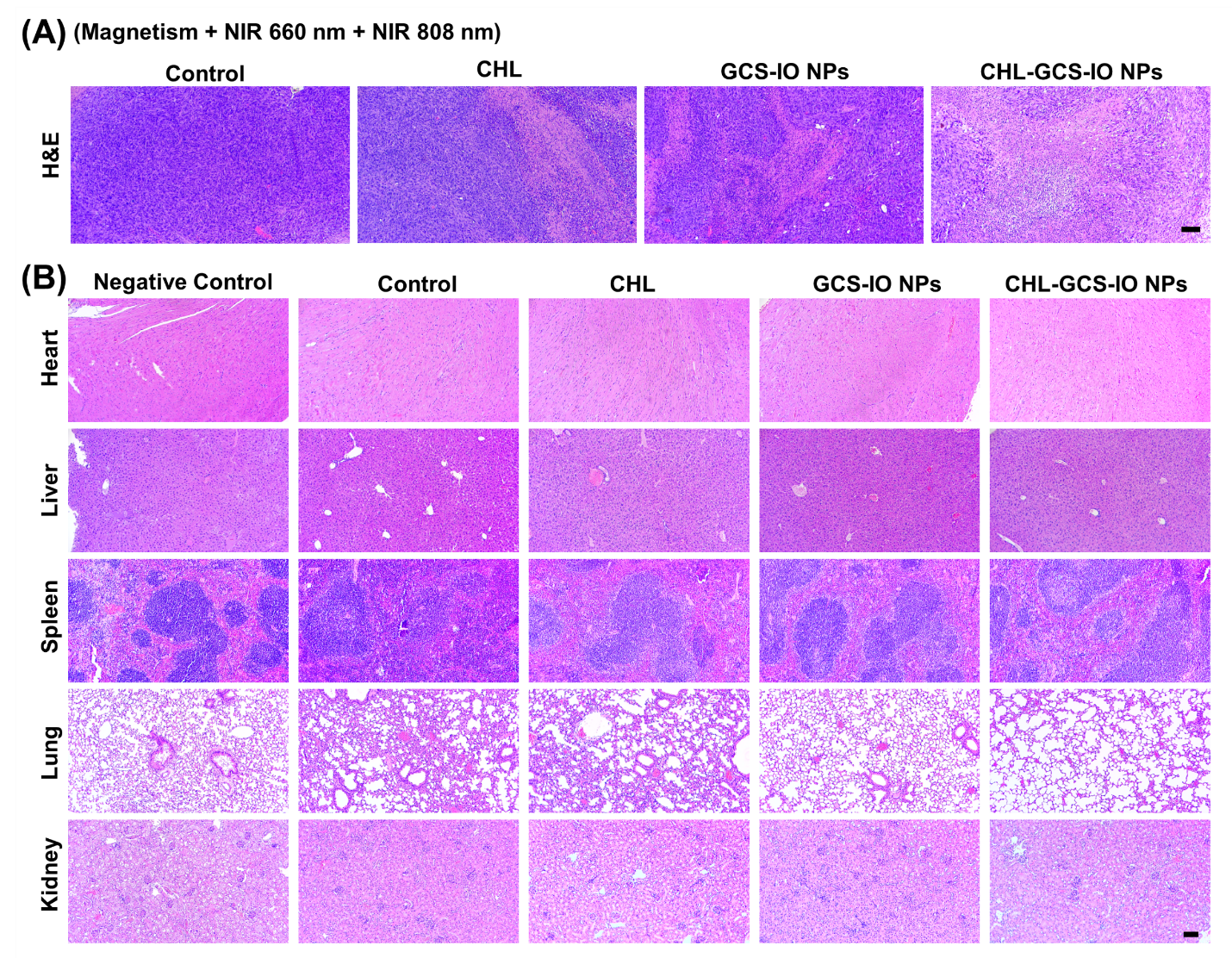


**Figure S2.** Histological analysis of tumor and major organs following treatment with *Chlorella* (CHL), glycol chitosan iron oxide nanoparticles (GCS-IO NPs), and CHL-GCS-IO NPs under magnetic targeting and near infrared (NIR) irradiation. (A) Hematoxylin and eosin (H&E) staining of tumor tissues. Representative images show the tumor histology in the control, CHL, GCS-IO NPs, and CHL-GCS-IO NPs treatment groups. The control group exhibited highly proliferative tumor cells with minimal necrosis. CHL-treated tumors displayed moderate necrosis but retained viable tumor regions, indicating limited therapeutic efficacy. The GCS-IO NPs group showed increased tumor necrosis, likely due to photothermal therapy (PTT) and ferroptosis, while the CHL-GCS-IO NPs group demonstrated widespread necrosis and significant tumor cell reduction, indicating potent therapeutic effects driven by photosynthesis therapy (PST), PTT, and magnetic targeting. Scale bar: 100 µm. (B) H&E staining of major organs (heart, liver, spleen, lungs, and kidneys). Scale bar: 100 µm.
